# A Mission-Oriented Flight Path and Charging Mechanism for Internet of Drones

**DOI:** 10.3390/s23094269

**Published:** 2023-04-25

**Authors:** Chenn-Jung Huang, Kai-Wen Hu, Hao-Wen Cheng, Yi-Sin Sie Lin

**Affiliations:** 1 Department of Computer Science & Information Engineering, National Dong Hwa University, Shoufeng, Hualien 974301, Taiwan; 2 Department of Electrical Engineering, National Dong Hwa University, Shoufeng, Hualien 974301, Taiwan

**Keywords:** drone, flight path and charging planning, data mining, machine learning, optimization

## Abstract

In addition to traditional battery exchange services and stationary charging stations, researchers have proposed wireless charging technology, such as decentralized laser charging or drone-to-drone charging in flight, to provide power to drones with insufficient battery electricity. However, the charging methods presented in the literature will inevitably cause drones to wait in line for charging during peak hours and disrupt their scheduled trips when the number of drones grows rapidly in the future. To the best of our knowledge, there have been no integrated solutions for drone flight path and charging planning to alleviate charging congestion, taking into account the different mission characteristics of drones and the charging cost considerations of drone operators. Accordingly, this paper provides adaptive charging options to help drone operators to solve the above-mentioned problems. Drones on ordinary missions can use conventional battery swap services, wired charging stations, or electromagnetic wireless charging stations to recharge their batteries as usual, whereas drones on time-critical missions can choose drone-to-drone wireless charging or decentralized laser charging deployed along the fight paths to charge the batteries of drones in flight. Notably, since fixed-wing drones have larger wing areas to install solar panels, they can also use solar energy to charge during flight if the weather is clear. The simulation results exhibited that the proposed work reduced the power load of the power grid during peak hours, met the charging needs of each individual drone during flight, and cut down the charging costs of drone operators. As a result, an all-win situation for drone operators, drone customers, and power grid operators was achieved.

## 1. Introduction

In recent years, drones have been widely used in civilian applications, such as search and rescue operations, road traffic monitoring, wildfire monitoring and remote sensing, and many other applications. The COVID-19 epidemic, which began in late 2019, has changed the way people shop for goods. Drones have become one of the most important transportation tools for e-commerce and logistics companies to deliver goods without human contact during the COVID-19 epidemic. In addition, the brilliant performance of drones in the Russia-Ukraine war starting in February 2022 has made them a key player in future military operations. With the rapid growth of logistics needs, the literature also predicts that drones will become a major mode of transportation in the logistics industry by 2040 [1]. In addition, Europe, the U.S., and Japan have been proposing air taxi services recently, and air taxi services will also become an important part of smart cities in the next few years. However, the short battery life of a drone limits its flight time, and it requires frequent battery charging to extend its battery life. Therefore, the battery life of drones will be another major challenge and a hot research topic in the industry before drones are widely used in the future [2].

Over the years, several studies have proposed different charging technologies to address the endurance problem of drone batteries [3]. In addition to the traditional battery swap service [4] and wired charging stations [5], some researchers have proposed the use of renewables to charge drone batteries. For example, Lv et al. proposed a charging scheme for drone batteries using the Lyapunov optimization technique [6]. The charging station used in this study is equipped with a facility that generates electricity from renewables to reduce the cost of purchasing electricity from the conventional power grid. However, Qin et al. reported in [7] that drone charging stations powered by renewables are susceptible to weather and capacity constraints of storage facilities. Thus, relying on renewables such as solar or wind power alone may cause the battery power supply for drones to fall short of expectations due to unstable weather conditions [8,9]. Although some studies have proposed the idea of using solar or wind power to extend the battery life of drones during flight [10], the possible shortage of drone battery power supplied by renewables due to climate change is still unresolved. It can be anticipated that more effective drone battery power supply technologies should be developed to maintain the required battery power for drone flight in a timely manner.

Currently, the battery life of a drone is mainly extended by using the above-mentioned traditional battery swap service and fixed-point charging stations. Since drones will be increasingly popular in the future, it is very likely that too many drones will need to charge their batteries at the same time during peak hours, causing congestion in battery exchange and fixed-point charging stations. The scheduled missions of drones will be affected just as traffic jams are observed during rush hours in metropolitan areas all over the world [11]. Therefore, if drones are used to transport urgent deliveries, battery exchange and fixed charging stations alone will not be able to meet the real-time needs of drone customers. On the contrary, the recently developed wireless power transfer technologies can provide wireless charging for drones at fixed points during flight or between flights to extend the battery life of drones. For example, Li et al. recently used a resonance coupling circuit to build a wireless charging system to help drones and continuously provide their battery power during flight [12]. The study includes a conventional wireless charging station that releases power only during the hovering charging period to reduce unnecessary power wastage of the wireless charging station. The study by Zhang et al. attempted to maintain stable current control of a drone hovering at a fixed wireless charging station during charging under the continuous fluctuation of coupling effect, disturbance of parameters, and variation of charging current [13]. In this study, an online trained radial basis function neural network was used to ensure the constant current output for battery charging. Rong et al. proposed a drone-to-drone wireless charging mechanism with high misalignment tolerance [14]. Their mechanism optimized the parameters of the transmission coil used for wireless charging using a simulated model and genetic algorithm. Gupta et al. proposed a wireless charging technique for charging drones that need electricity during flight [15]. Game theory was employed to establish a tariff mechanism for the electricity market.

In terms of wireless transmission technology for non-electromagnetic signals, the development of laser charging technology has attracted the most attention. Mohammadnia et al. used a laser beam of specific wavelength and frequency to power a PV cell installed on a drone and evaluated the effectiveness of the laser charging mechanism [16]. Their study showed that the laser charging mechanism can effectively extend the battery life of the drone. Recently, researchers proposed the distributed laser charging mechanism [17]. This mechanism uses intracavity laser technology, which can automatically charge drones without specific tracking and positioning as long as the transceivers are within each other’s line of sight [18]. Notably, the decentralized laser power transmission technology is safer and can transmit power over longer distances because the power transmission will be stopped if an object blocks the laser light in the line of sight during wireless charging. Kim and Lim proposed a dynamic wireless charging system [19], which deploys wireless charging devices along the flight path and provides power to drones with insufficient battery life through wireless charging during flight.

In addition to the ongoing innovation of wireless charging technologies, researchers have also started to focus on the overall planning of drone flight routes and battery charging in recent years. Zhao et al. considered the efficiency of charging stations and wireless power transmission to drones simultaneously when planning drone flight routes [20]. In contrast to the traditional alternating optimization approach, this study adopted the concave-convex procedure and the penalty dual decomposition to solve the optimization problem of flight path and charging planning. Kilic et al. established a grid architecture for drone charging stations and integrated the grids with flight route planning [21]. They proposed the shortest path algorithm that fits the grid characteristics of the charging stations. Wang et al. designed a drone path planning and battery charging scheme to guide the drone to a suitable charging station for battery charging based on the user’s location [22]. Their study converted the optimization problem into an integer linear program and developed a recursive algorithm to reduce the computational complexity of the optimization process. Alyassi et al. treated the flight route and charging planning of drones as a multi-criteria asymmetric traveling salesman problem [23]. The optimization objective is the overall trip consumption time of the drone, including charging time. To simplify the computation of the optimization process, their study also proposed a sub-optimal algorithm to develop the flight task and charging strategy. Arafat et al. proposed a cargo delivery flight route and charging planning for drones by first clustering the customers according to the delivery area [24]. They then cut the flight route into segments according to the safe flight distance and employed mixed-integer linear planning to solve the route planning problem. Pinto and Lagorio derived a mathematical model for the flight route and charging planning for drone cargo delivery [25]. The number of deployed drone charging stations and the drone flight distance were chosen as the optimization objectives. Ribeiro et al. extended the traditional vehicle routing problem by deploying mobile charging stations to support drone search and rescue missions [26]. Genetic algorithms were used in their approach to solve the flight route and charging planning optimization problem. Oubbati et al. proposed a drone flight path and drone-to-drone charging planning algorithm [27]. Their algorithm applied deep reinforcement learning to determine the rendezvous point of the drones and optimize the flight paths of the drones, as well.

With the rapid growth in the number of drones, either traditional battery swap stations and fixed-point wired/wireless charging stations will definitely cause drones to queue up for charging during peak hours and affect their scheduled trips when drones are widely used [27]. From the above-mentioned literature, it can be seen that several studies have been conducted on the use of wireless charging technologies to provide power to drones with insufficient battery life, such as decentralized laser charging or drone-to-drone charging that allows battery charging of a drone in flight. However, due to the different mission characteristics and needs of drones, it is essential to stipulate an effective charging policy according to the mission needs of drones, so that drones performing time-critical missions can fly to their destinations in time to complete their missions according to the established plan. In the meantime, the drone operators can cut down the charging cost for the drones that perform ordinary missions, such as e-commerce and logistics.

To the best of our knowledge, no literature has proposed the above-mentioned charging strategy based on the mission characteristics of drones and the cost of charging, and this work thus proposes an integrated solution to alleviate the congestion of drone flight routes and charging plans. The drones can arrive at their destinations in time to complete their missions. Drones on ordinary missions can use conventional battery swap services, wired charging stations, or electromagnetic signal-delivery wireless charging stations to recharge their batteries; however, drones on urgent missions with time-critical requirements can choose to use drone-to-drone in-flight wireless charging or the above-mentioned decentralized laser charging deployed along the fight paths to provide power to drones with insufficient battery life. In addition, drones can be divided into rotary-wing and fixed-wing drones, and a fixed wing drone has a larger wing area to install solar panels. Accordingly, a fixed-wing drone can also use solar power to charge during flight if the weather is clear [10]. The dynamic soaring technology [9], which is commonly seen in the literature, can also be extended from this work to provide battery power for drones.

The main layout of this paper is as follows. Section 2 is the algorithm proposed in this work. The simulation results, analysis, and discussion will appear in Section 3. Finally, the conclusion and future work will be given in Section 4.

## 2. Flight Path and Charging Mechanism for Internet of Drones

**Figure 1:**
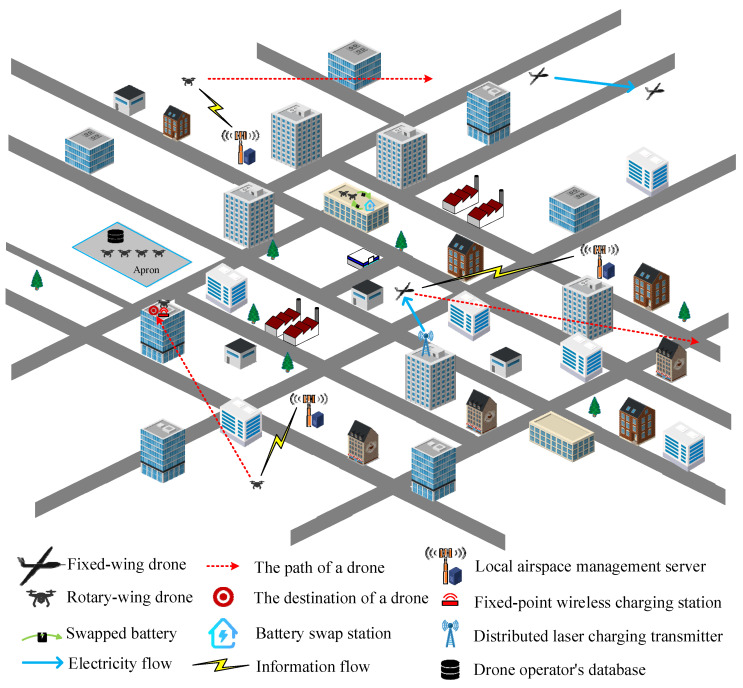
Flight path and charging planning for the Internet of drones in a sample scenario.

In this work, the flight route of a drone is planned before takeoff. The whole airspace is cut into individual local airspaces according to the geographical area and altitude, and each local airspace is further cut into basic air-cubes and combined into an air-matrix. Each air-cube can only allow one aircraft to pass through it at each time spot, so the flight paths of all aircraft must be spaced apart to avoid collisions. In order to reduce the complexity of the calculation, a local airspace management server as shown in Figure 1 is set up in each local airspace to record the time when all aircraft pass through the air-cubes under its jurisdiction. Drone operators provide low-cost battery swap services or fixed-point wired/wireless charging stations on the aprons to charge the drones’ batteries while they are idle. In the event of a state of charge (SoC) shortage during flight, different charging options can be selected to charge the drone’s battery on the way to its destination. Drones are divided into two categories according to their mission characteristics, including ordinary missions and time-critical missions. An ordinary mission does not have a strict mission completion time, just like the current operation mode of ordinary parcel delivery service, so the charging option of either fixed point charging or battery swap service nearby can be chosen for drone charging. On the other hand, drones on time-critical missions, which need to complete tasks in time, are given priority to decentralized laser charging facilities along some routes or drone-to-drone in-flight wireless charging options to charge the batteries of drones on time-critical missions with insufficient battery storage.

**Figure 2:**
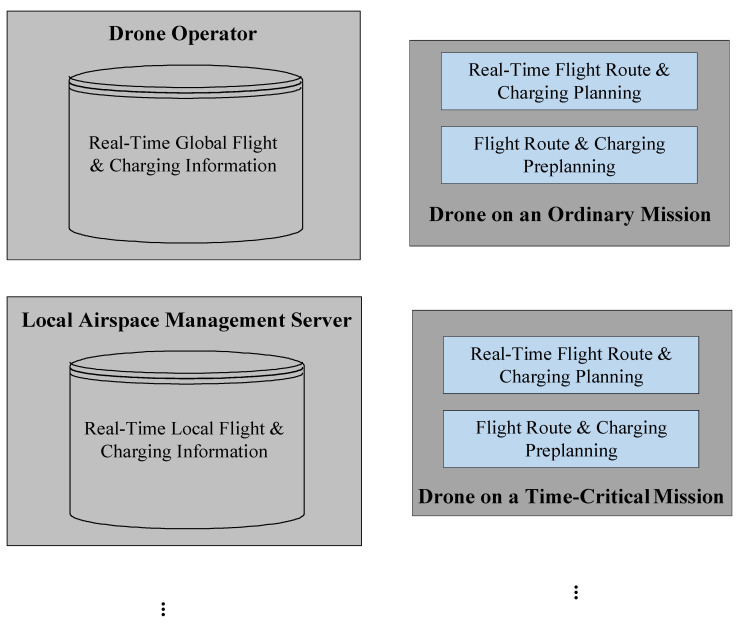
Schematic diagram of the proposed algorithm.

After assigning a drone to an ordinary mission, the drone operator activates the “Flight Route and Charging Preplanning Module” for a drone on an ordinary mission in the upper right of Figure 2 to plan the flight route of the drone. For a drone assigned to a time-critical mission, the “Flight Route and Charging Preplanning Module” for a drone on a time-critical mission in the lower right of Figure 2 is activated to plan the flight route. The conflict-free A* algorithm proposed in [28] avoids fixed obstacles or other moving obstacles, such as an inflight drone, and calculates the shortest flight route for drones. Accordingly, this work focuses on the individual charging requirements of drones and employs the algorithm proposed in [28] to filter the flight routes and charging methods that suit the mission characteristics and charging cost considerations of drones. Notably, the three vertical dots under the two above-mentioned modules in Figure 2 are used to indicate that the two modules are used by drones on ordinary and time-critical missions for flight path and charging planning, respectively.

The two modules mentioned above first use the algorithm of [28] to calculate the shortest flight path to the destination, and the flight path planning will also confirm whether the drone’s battery has enough power to reach the destination. If the drone runs out of battery power before reaching its destination, the drone on an ordinary mission will choose either a battery swap service to replace the drone’s battery or a fixed wireless or wired charging station to recharge the drone’s battery during the flight path to the destination. Once the flight route and charging point are determined, the drone will follow the planned route to the charging point to recharge the drone’s battery. Since the drone may arrive at each local airspace during peak hours due to the uncertainty of the charging waiting time, the drones on ordinary missions will activate the “Flight Route and Charging Preplanning Module” to re-plan the flight route from the charging point to the next charging point or destination after its battery charging is completed. If a drone on a time-critical mission needs to be recharged, it can make use of the distributed laser charging facilities deployed on the flight route. If the distributed laser charging facilities are not close to its fight route, the drone on ordinary missions or idle on the aprons will be checked to see whether it has spare power to provide power to a drone on a time-critical mission via drone-to-drone inflight charging. The two approaches are chosen in this work to minimize the delay of charging for the drones on time-critical missions. The corresponding local airspace management server will also update the air traffic and charging information of the managed local airspaces after the drone has completed its flight route and charging plan. The battery capacity of each drone that supports a drone on a time-critical mission while traveling through each airspace block is updated, as well.

It is well known that ambulances are given priority in the use of roads during their missions. Similar to this practice, this work allows drones on time-critical missions to have priority in the use of airspaces. Therefore, during the flight of a drone on an ordinary mission, each local airspace originally planned for the flight may be used by a drone on a time-critical mission. In addition, the flight speed of the drone may be slowed down due to some climate problem, and the airspaces originally planned for the flight might be occupied during the late arrival of the drone. In this case, the drone must change its flight path or delay its arrival at the originally planned airspace. The “Real-Time Flight Route and Charging Planning Module” for a drone on an ordinary mission in the upper right of Figure 2 is used to plan the drone’s new flight route in real time based on the updated air traffic and charging-related information of local airspaces. If charging is required during the flight, the drone’s battery can be charged at a nearby charging station or battery swap service facility when changing the flight route.

Drones on time-critical missions might also be affected by climate change during flight. In this case, the “Real-Time Flight Route and Charging Planning Module” for a drone on a time-critical mission in the lower right of Figure 2 will be used to modify the flight route for the affected drones. In this work, it is assumed that the drone operator will set up a real-time flight and charging information database for the global airspace, and each local airspace management server will inform the drone operator of the latest flight and charging information of each local airspace in real time, which will be used as the basis for drone route and charging planning. In addition, if a drone on an ordinary mission or idle on an apron needs to support battery power with a drone on a time-critical mission, this module will also modify the route of the drone supporting power to ensure that both drones can fly in sequence during drone-to-drone inflight charging. If a drone on a time-critical mission prefers a drone-to-drone inflight wireless charging option, this module will send a request for drone-to-drone charging to each local airspace segment management server along the drone’s flight route. Upon receipt of the request, the local airspace segment management servers of the selected flight route will assist in searching for a drone on an ordinary mission or an idle drone that can arrive earlier and provide extra power, based on the arrival time of the requesting drone with power requirements. Once any candidate drones are found, the requesting drone then selects a flight route based on the electricity price of the candidate drones providing power, considering the arrival time and charging cost. Since the arrival time of the drones at each local airspace may be inconsistent with the original estimate due to weather conditions, the drone providing power is required to maintain a fixed distance from the requesting drone during the flight after they arrive at the given airspaces. Therefore, the module will also update the flight path of the drone providing power to ensure that it can be wirelessly charged in synchronization with the drone requiring power. This work assumes that transportation organizations or power operators will provide subsidies for the installation of wireless charging facilities for drones, so as to attract more drone operators to provide drones with excess power to charge the batteries of drones on time-critical missions in a timely manner.

The following subsections describe the detailed steps of each module shown in Figure 2.

### 2.1. Flight Route and Charging Preplanning for a Drone on an Ordinary Mission

After determining the departure time of a drone on an ordinary mission, this module is activated before takeoff to plan the drone’s flight route. As mentioned earlier, the conflict-free A* algorithm proposed in [28] avoids obstacles such as other inflight drones and calculates the shortest flight route for the drone. After the flight route is determined, this module informs the local airspace management servers of their governing air-cubes that the drone passes through. Notably, the conflict-free A* algorithm here also takes into account the hovering characteristics of the rotary-wing drones, and flexibly adjusts the drones’ traverse time through each local airspace to avoid collision with other drones. The fixed-wing drones cannot hover at waypoints during flight due to their minimum speed constraints.

If the drone battery is not sufficient to reach the destination, this module will consider the cost of charging the drone’s battery and the time to reach the destination to choose the appropriate charging option and flight route. As aforementioned, the charging options adopted for a drone on an ordinary mission in this work are fixed-point wireless charging stations and battery swap services that require a time-consuming wait for charging. However, the waiting time for a drone recharging at charging points, such as fixed-point wireless charging stations and battery swap services, during peak hours is uncertain. If a drone needs to be recharged during the flight, this module will reschedule the subsequent flight segment(s) after the drone finishes recharging its battery at a designated charging point. Once the next flight segment of the drone is confirmed, this module will inform the local airspace management server that governs the local airspaces the drone flies through. In addition, because solar panels can be installed on the large wing area of a fixed-wing drone, this module will use solar energy to charge a fixed-wing drone in case of sunny weather during flight. Notably, this module can also be extended to use wind power or other innovative renewable charging technologies to charge drones in the future.

The detailed steps of this module are as follows.

Step 1: Based on the latest airspace information stored in the drone operators’ database and the flight path cost in terms of the time for the drone to arrive at the destination from the departure location, the flight path of the drone is estimated using the conflict-free A* algorithm proposed in [28] as follows:

(1) $$R^{d}=\left[\left(a_{0}^{d},a_{1}^{d}\right),\left(a_{1}^{d},a_{2}^{d}\right),\cdots ,\left(a_{i}^{d},a_{i+1}^{d}\right),\cdots ,\left(a_{m^{d}-1}^{d},a_{m^{d}}^{d}\right)\right],$$

where $a_{0\;}^{d}$ and $a_{m^{d}}^{d}$ represent the indices of the air-cube at the departure point and the destination of drone *d* on an ordinary mission, respectively, and $a_{i}^{d}$ is the index of the *i*th air-cube along the route.

Step 2: Estimate whether the battery power consumed by the drone’s flight path is sufficient to reach its destination by:

(2) $$\begin{array}{ll} {SoC}_{a_{0}^{d}}^{d}-{rw}^{d}\cdot & \sum_{i=0}^{m^{d}-1}\left[{RAP}^{d}\left({\vec{av}}_{a_{i}^{d},a_{i+1}^{d}}^{d}\right)\cdot {ft}_{a_{i}^{d},a_{i+1}^{d}}^{d}+{RAP}^{d}\left(0\right)\cdot {ht}_{a_{i}^{d}}^{d}\right]\\ & -\left(1-{rw}^{d}\right)\\ & \cdot \left\{\sum_{i=0}^{m^{d}-1}\left[{FAP}^{d}\left({\vec{av}}_{a_{i}^{d},a_{i+1}^{d}}^{d},{ft}_{a_{i}^{d},a_{i+1}^{d}}^{d}\right)\cdot {ft}_{a_{i}^{d},a_{i+1}^{d}}^{d}\right]+{tlt}^{d}\right\}\\ & \geq \left(1-{rw}^{d}\right)\cdot \eta^{d}\cdot \sum_{i=0}^{m^{d}-1}S_{a_{i}^{d},a_{i+1}^{d}}^{d}\left({at}_{a_{i}^{d}}^{d}\right)\\ & \cdot \left[{SAP}^{d}\left({tp}_{a_{i}^{d},a_{i+1}^{d}}^{d}\left({at}_{a_{i}^{d}}^{d}\right),{si}_{a_{i}^{d},a_{i+1}^{d}}^{d}\left({at}_{a_{i}^{d}}^{d}\right)\right)\cdot {ft}_{a_{i}^{d},a_{i+1}^{d}}^{d}\right]\\ & +{\underline{SoC}}^{d}, \end{array}$$

(3) $$at_{a_{i+1}^{d}}^{d}=at_{a_{i}^{d}}^{d}+ft_{a_{i}^{d},a_{i+1}^{d}}^{d}+rw^{d}\cdot ht_{a_{i}^{d}}^{d},\;\;0\leq i<m^{d},$$

(4) $$ft_{a_{i}^{d},a_{i+1}^{d}}^{d}=\frac{\left\|{\vec{bl}}_{a_{i}^{d},a_{i+1}^{d}}\right\|}{\left\|{\vec{av}}_{a_{i}^{d},a_{i+1}^{d}}^{d}\right\|},\;\;0\leq i<m^{d},$$

where $SoC_{a_{0}^{d}}^{d}$ and ${\underline{SoC}}^{d}$ represent the remaining battery power and the lower limit of battery power at the departure point of drone *d*, respectively. ${\vec{bl}}_{a_{i}^{d},a_{i+1}^{d}}$ denotes the distance vector between the center of the *i*th air-cube $a_{i}^{d}$ and the center of the (*i*+1)th air-cube $a_{i+1}^{d}$ of the flight route of drone *d*. $RAP^{d}\left(\cdot \right)$ and $FAP^{d}\left(\cdot \right)$ stand for the battery power consumption functions derived from [29,30] for rotary-wing and fixed-wing drones, respectively. $tlt^{d}$ is the battery power consumption for fixed-wing drones during take-off and landing, which is derived in [30]. ${\vec{av}}_{a_{i}^{d},a_{i+1}^{d}}^{d}$ denotes the full speed vector of drone *d* from $a_{i}^{d}$ to $a_{i+1}^{d}$ based on the maximum horizontal and vertical flight speeds of the drone provided by the drone operator. $ft_{a_{i}^{d},a_{i+1}^{d}}^{d}$ represents the time spent from $a_{i}^{d}$ to $a_{i+1}^{d}$, and $ht_{a_{i}^{d}}^{d}$ is the hovering time of the rotary-wing drone at $a_{i}^{d}$. Here the values of ${\vec{bl}}_{a_{i}^{d},a_{i+1}^{d}}$, ${\vec{av}}_{a_{i}^{d},a_{i+1}^{d}}^{d}$, and $ht_{a_{i}^{d}}^{d}$ can be obtained during the calculation of the flight path by the conflict-free A* algorithm. $at_{a_{i}^{d}}^{d}$ is the time when the drone reaches $a_{i}^{d}$, and the binary flag $S_{a_{i}^{d},a_{i+1}^{d}}^{d}\left(at_{a_{i}^{d}}^{d}\right)=1$ implies that the weather is clear at the time $at_{a_{i}^{d}}^{d}$ that the drone flies between $a_{i}^{d}$ and $a_{i+1}^{d}$, and it is suitable for the fixed-wing drone to get charged with solar panels. $SAP^{d}\left(\cdot \right)$ is the charging power function via solar power [10], whose parameters $tp_{a_{i}^{d},a_{i+1}^{d}}^{d}\left(at_{a_{i}^{d}}^{d}\right)$ and $si_{a_{i}^{d},a_{i+1}^{d}}^{d}\left(at_{a_{i}^{d}}^{d}\right)$ represent air temperature and solar irradiance at the time $at_{a_{i}^{d}}^{d}$ that the drone flies from $a_{i}^{d}$ to $a_{i+1}^{d}$, respectively. $\eta^{d}$ is the charging efficiency of the drone’s battery, and its value is a positive number smaller than one.

Step 3: If the drone’s battery power is sufficient to reach the destination, calculate the extra battery power $ee_{a_{i}^{d}}^{d}$ at $a_{i}^{d}$ that the drone can provide other drones on time-critical missions using the following equation, and then proceed to Step 6.

(5) $$ee_{a_{i}^{d}}^{d}=SoC_{a_{m^{d}}^{d}}^{d}-{\underline{SoC}}^{d},0\leq i<m^{d}$$

Instead, when the battery power of the drone is not sufficient to reach the destination, this module requests the local airspace management servers along the route calculated in Step 1 and the local airspace management servers surrounding the route for the latest traffic information of the local airspaces they manage. Then, proceed to the next step to find a suitable charging point to charge the battery of drone *d*.

Step 4: Based on the flight path $R^{d}$ obtained in Step 1, select the nearest $K^{d}$ charging points to any of the air-cubes along the route $R^{d}$. The distance is estimated as follows.

(6) $$dist_{R^{d},\gamma }=\frac{{\vec{bl}}_{a_{0}^{d},\gamma }\cdot {\vec{bl}}_{a_{0}^{d},a_{m^{d}}^{d}}}{∥{\vec{bl}}_{a_{0}^{d},a_{m^{d}}^{d}}∥},$$

where *γ* is the index of the air-cube where the charging point is located.

Step 5: Select the charging points suitable for drone *d* using the following equations.

(7) $$\underset{L^{d}}{\arg\min}\left\{\omega_{1}^{d}\cdot \left[\phi_{p_{l,n_{l}^{d}}^{d}}^{d}\cdot {RP}_{p_{l,n_{l}^{d}}^{d}}^{d}\left({at}_{p_{l,n_{l}^{d}}^{d}}^{d}\right)\cdot {pcp}_{p_{l,n_{l}^{d}}^{d}}^{d}\cdot {ct}_{p_{l,n_{l}^{d}}^{d}}^{d}+\Psi_{p_{l,n_{l}^{d}}^{d}}^{d}\;\;\;\;\;\;\;\;\;\;\cdot {RP}_{p_{l,n_{l}^{d}}^{d}}^{d}\left({at}_{p_{l,n_{l}^{d}}^{d}}^{d}\right)\cdot \left({\bar{SoC}}^{d}-{SoC}_{p_{l,n_{l}^{d}}^{d}}^{d}\right)\right]+\omega_{2}^{d}\;\;\;\;\;\;\;\;\;\;\;\;\cdot \sum_{0\leq l\leq L^{d}}\sum_{0\leq i<p_{l,n_{l}^{d}}^{d}}\left\|{\vec{bl}}_{p_{l,i}^{d},p_{l,i+1}^{d}}\right\|+\omega_{3}^{d}\cdot \left(a_{m^{d}}^{d}-{at}_{a_{0}^{d}}^{e}\right)\right\},$$

subject to:

(8) $$1\leq L^{d}\leq K^{d}+1,$$

(9) $$P_{l}^{d}=\left[\left(p_{l,0}^{d},p_{l,1}^{d}\right),\left(p_{l,1}^{d},p_{l,2}^{d}\right),\cdots ,\left(p_{l,i}^{d},p_{l,i+1}^{d}\right),\cdots ,\left(p_{l,n_{l}^{d}-1}^{d},p_{l,n_{l}^{d}}^{d}\right)\right],1\leq l\leq L^{d}$$

(10) $$p_{0,0}^{d}=a_{0}^{d},\;p_{L^{d},n_{L^{d}}^{d}}^{d}=a_{m^{d}}^{d}$$

(11) $$p_{l+1,0}^{d}=p_{l,n_{l}^{d}}^{d},\;1\leq l<L^{d}$$

(12) $$SoC_{p_{l,0}^{d}}^{d}>{\underline{SoC}}^{d}+\left(1-rw^{d}\right)\cdot tlt^{d},\;1\;\leq \;l\;\leq \;t^{d}$$

(13) $$ft_{p_{l,i}^{d},p_{l,i+1}^{d}}^{d}=\frac{\left\|{\vec{bl}}_{p_{l,i}^{d},p_{l,i+1}^{d}}\right\|}{\left\|{\vec{av}}_{p_{l,i}^{d},p_{l,i+1}^{d}}^{d}\right\|},\;\;1\leq l\leq L^{d},\;0\leq i<n_{l}^{d}$$

(14) $${{SoC}_{p_{l,i+1}^{d}}^{d}=SoC}_{p_{l,i}^{d}}^{d}-{rw}^{d}\cdot \left[{RAP}^{d}\left({\vec{av}}_{p_{l,i}^{d},p_{l,i+1}^{d}}^{d}\right)\cdot {ft}_{p_{l,i}^{d},p_{l,i+1}^{d}}^{d}+{{RAP}^{d}\left(0\right)\cdot ht}_{p_{l,i}^{d}}^{d}\right]-\;\left(1-{rw}^{d}\right)\cdot {FAP}^{d}\left({\vec{av}}_{p_{l,i}^{d},p_{l,i+1}^{d}}^{d},{ft}_{p_{l,i}^{d},p_{l,i+1}^{d}}^{d}\right)\cdot {ft}_{p_{l,i}^{d},p_{l,i+1}^{d}}^{d}>\left(1-{rw}^{d}\right)\cdot \eta^{d}\cdot \;S_{p_{l,i}^{d},p_{l,i+1}^{d}}^{d}\left({at}_{p_{l,i}^{d}}^{d}\right)\cdot {SAP}^{d}\left({tp}_{p_{l,i}^{d},p_{l,i+1}^{d}}^{d}\left({at}_{p_{l,i}^{d}}^{d}\right),{si}_{p_{l,i}^{d},p_{l,i+1}^{d}}^{d}\left({at}_{p_{l,i}^{d}}^{d}\right)\right)\cdot {ft}_{p_{l,i}^{d},p_{l,i+1}^{d}}^{d}+\;{\underline{SoC}}^{d},\;\;\;\;\;\;1\leq l\leq L^{d},\;0\leq i<n_{l}^{d}$$

(15) $$at_{p_{l,i+1}^{d}}^{d}=at_{p_{l,i}^{d}}^{d}+ft_{p_{l,i}^{d},p_{l,i+1}^{d}}^{d}+rw^{d}\cdot ht_{p_{l,i}^{d}}^{d},1\leq l\leq L^{d},\;0\leq i<n_{l}^{d}$$

(16) $$at_{p_{l+1,0}^{d}}^{d}=at_{p_{l,n_{l}^{d}}^{d}}^{d}+\phi_{p_{l,n_{l}^{d}}^{d}}^{d}\cdot ct_{p_{l,n_{l}^{d}}^{d}}^{d}+\Psi_{pl,n_{l}^{d}d}^{d}\cdot bst_{p_{l,n_{l}^{d}}^{d}}^{d}+WT_{p_{l,n_{l}^{d}}^{d}}^{d}\left(at_{p_{l,n_{l}^{d}}^{d}}^{d}\right),\;\;\;\;\;\;\;\;\;\;1\leq l<L^{d}$$

(17) $${}_{p_{l,n_{l}^{d}}^{d}}^{d}\cdot \left(\eta^{d}\cdot pcp_{p_{l,n_{l}^{d}}^{d}}^{d}\cdot ct_{p_{l,n_{l}^{d}}^{d}}^{d}+SoC_{p_{l,n_{l}^{d}}^{d}}^{d}\right)\leq {\bar{SoC}}^{d},1\leq l\leq L^{d}$$

(18) $$\phi_{p_{l,n_{l}^{d}}^{d}}^{d}+\Psi_{pl,n_{l}^{d}d}^{d}\;=\;1,1\leq l\leq L^{d}$$

(19) $$ee_{p_{l,i}^{d}}^{d}=SoC_{p_{l,n_{l}^{d}}^{d}}^{d}-{\underline{SoC}}^{d},\;1\;\leq \;l\;\leq \;L^{d},0\;\leq \;i\;\leq \;n_{l}^{d}$$

The parameters used in the above equations are defined as follows:

(i) The three parameters from the left to the right in the calculation of arg min(·) represent the charging cost of drones’ batteries, the flight distance from the departure point to the destination, and the flight time required for the drone to reach the destination. The drone operator can flexibly adjust the weights of $\omega_{1}^{d}$, $\omega_{2}^{d}$, and $\omega_{3}^{d}$ according to the mission characteristics and operating cost considerations.
(ii) $L^{d}$ is the number of segments that drone *d* travels and charges the battery via $L^{d}-1$ charging point. $P_{l}^{d}$ is the *l*th segment of the whole flight path of drone *d* as calculated by the conflict-free A* algorithm from [28]. $at_{a_{0}^{d}}^{d}$ and $at_{p_{l,n_{l}^{d}}^{d}}^{d}$ represent the takeoff time of the drone at the departure point $a_{0}^{d}$ and the arrival time at the charging point $p_{l,n_{l}^{d}}^{d}$*,* respectively.
(iii) The binary flags ${}_{p_{l,n_{l}^{d}}^{d}}^{d}$ and ${}_{p_{l,n_{l}^{d}}^{d}}^{d}$ are used to indicate whether the drone charges the battery at a fixed-point charging station or a battery exchange service facility located at $p_{l,n_{l}^{d}}^{d}$, respectively. The binary flag for the charging option is set to one if the option is chosen to charge the drone battery, otherwise it is zero. ${\bar{SoC}}^{d}$ represents the upper limit of the drone’s battery capacity, and $SoC_{p_{l,n_{l}^{d}}^{d}}^{d}$ represents the remaining capacity of the drone when it reaches the charging point at $p_{l,n_{l}^{d}}^{d}$.
(iv) $RP_{p_{l,n_{l}^{d}}^{d}}^{d}\left(at_{p_{l,n_{l}^{d}}^{d}}^{d}\right)$ is the real-time charging cost when the drone arrives at the charging point located at $p_{l,n_{l}^{d}}^{d}$. $pcp_{p_{l,n_{l}^{d}}^{d}}^{d}$ denotes the charging power at the fixed-point charging station located at $p_{l,n_{l}^{d}}^{d}$, $ct_{p_{l,n_{l}^{d}}^{d}}^{d}$ stands for the actual charging time of the drone’s battery at the fixed-point charging station located at $p_{l,n_{l}^{d}}^{d}$, and ${bst}_{p_{l,n_{l}^{d}}^{d}}^{d}$ is the battery swap time of the drone’s battery at the battery swap service. $WT_{p_{l,n_{l}^{d}}^{d}}^{d}\left(at_{p_{l,n_{l}^{d}}^{d}}^{d}\right)$ is the waiting time spent at the fixed-point charging station or the battery swap service facility located at $p_{l,n_{l}^{d}}^{d}$. This module estimates the value of $WT_{p_{l,n_{l}^{d}}^{d}}^{d}\left(at_{p_{l,n_{l}^{d}}^{d}}^{d}\right)$ based on the charging point history data using the support vector regression (SVR) technique.
(v) $RAP^{d}\left(\cdot \right)$ and $FAP^{d}\left(\cdot \right)$ represent the battery power functions derived from [29,30] for rotary-wing and fixed-wing drones, respectively. ${\vec{bl}}_{p_{l,i}^{d},p_{l,i+1}^{d}}$ stands for the distance vector of the drone from the center of the *i*th air-cube $p_{l,i}^{d}$ to the center of the (*i*+1)th air-cube $p_{l,i+1}^{d}$, ${\vec{av}}_{p_{l,i}^{d},p_{l,i+1}^{d}}^{d}$ is the full speed vector of the drone flying from $p_{l,i}^{d}$ to $p_{l,i+1}^{d}$, $ft_{a_{i}^{d},a_{i+1}^{d}}^{d}$ represents the flying time of the drone from $p_{l,i}^{d}$ to $p_{l,i+1}^{d}$, $tlt^{d}$ is the power consumption during the takeoff and landing of the fixed-wing drone, and $ht_{p_{l,i}^{d}}^{d}$ represents the hovering time of the rotary-wing drone at $p_{l,i}^{d}$. The binary flag $S_{p_{l,i}^{d},p_{l,i+1}^{d}}^{d}\left(at_{p_{l,i}^{d}}^{d}\right)$ = 1 represents if the weather is clear at the time $at_{p_{l,i}^{d}}^{d}$ and it is suitable for the fixed-wing drone with solar panels to charge with solar energy. $SAP^{d}\left(\cdot \right)$ is the solar charging power function [10], whose parameters $tp_{p_{l,i}^{d},p_{l,i+1}^{d}}^{d}\left(at_{p_{l,i}^{d}}^{d}\right)$ and $si_{p_{l,i}^{d},p_{l,i+1}^{d}}^{d}\left(at_{p_{l,i}^{d}}^{d}\right)$ stand for air temperature and solar irradiance at the time $at_{p_{l,i}^{d}}^{d}$.
(vi) $ee_{p_{l,i}^{d}}^{d}$ represents the extra battery power of drone *d* in $p_{l,i}^{d}$ that the drone can offer to other drones. When $ee_{p_{l,i}^{d}}^{d}$ is not zero, it is used to indicate that the drone is categorized as a drone that performs an ordinary mission. Since drones on time-critical missions have priority to use the air-cubes allocated to the drones on ordinary missions, the local airspace management server will update its database when drone *d* is requested to yield air-cube $p_{l,i}^{d}$ to a drone on a time-critical mission. Notably, the flight path of drone *d* that yields the air-cubes will be adjusted in the next subsection after a drone on a time-critical mission issues a request to use the air-cube assigned to drone *d*.

Step 6: After determining the flight path of drone *d*, information about the flight and charging of drone *d* is transmitted to the corresponding local airspace management servers.

### 2.2. Flight Route and Charging Preplanning for a Drone on a Time-Critical Mission

As aforementioned, drones on ordinary missions plan their flight routes before takeoff and notify the local airspace management server of the air-cubes the drones will fly through and the excess battery storage capacity at each air-cube along the route. In this module, drones on time-critical missions are allowed to prioritize the use of air-cubes that the drones on ordinary missions will pass through. This module will also adjust the flight path of the drones on ordinary missions after they are forced to yield the air-cubes. Additionally, if the battery of a drone on a time-critical mission needs to be charged during flight, in addition to choosing a route with a decentralized laser charging station, a drone-to-drone wireless charging option can also be used to allow a drone on an ordinary mission to provide power to the requesting drone by using radio frequency wireless power transmission technology during flight.

The detailed steps of this module are as follows.

Step 1: Based on the latest flight and charging-related airspace information recorded by the drone operators and the traverse time from the departure location to the destination as the cost of the flight path, the flight path of the drone performing a time-critical mission is estimated using the conflict-free A* algorithm proposed in [28] as follows.

(20) $$R^{e}=\left[\left(a_{0}^{e},a_{1}^{e}\right),\left(a_{1}^{e},a_{2}^{e}\right),\cdots ,\left(a_{i}^{e},a_{i+1}^{e}\right),\cdots ,\left(a_{m^{e}-1}^{e},a_{m^{e}}^{e}\right)\right],$$

where $a_{0}^{e}$ is the index of the air-cube of the departure point of drone *e*, $a_{m^{e}}^{e}$ is the index of the air-cube of the destination, and $a_{i}^{e}$ is the index of the *i*th air-cube along the route.

Step 2: Examine whether the battery power consumed by the drone’s flight is sufficient to reach its destination.

(21) $$\begin{array}{ll} {SoC}_{a_{0}^{e}}^{e}-{rw}^{e}\cdot & \sum_{i=0}^{m^{e}-1}\left[{RAP}^{e}\left({\vec{av}}_{a_{i}^{e},a_{i+1}^{e}}^{e}\right)\cdot {ft}_{a_{i}^{e},a_{i+1}^{e}}^{e}+{RAP}^{d}\left(0\right)\cdot {ht}_{a_{i}^{e}}^{e}\right]-\left(1-{rw}^{e}\right)\\ & \cdot \left\{\sum_{i=0}^{m^{e}-1}\left[{FAP}^{e}\left({\vec{av}}_{a_{i}^{e},a_{i+1}^{e}}^{e},{ft}_{a_{i}^{e},a_{i+1}^{e}}^{e}\right)\cdot {ft}_{a_{i}^{e},a_{i+1}^{e}}^{e}\right]+{tlt}^{e}\right\}\\ & \geq \left(1-{rw}^{e}\right)\cdot \eta^{e}\\ & \cdot \sum_{i=0}^{m^{e}-1}S_{a_{i}^{e},a_{i+1}^{e}}^{e}\left({at}_{a_{i}^{e}}^{e}\right)\cdot {SAP}^{e}\left({tp}_{a_{i}^{e},a_{i+1}^{e}}^{e}\left({at}_{a_{i}^{e}}^{e}\right),{si}_{a_{i}^{e},a_{i+1}^{e}}^{e}\left({at}_{a_{i}^{e}}^{e}\right)\right)\\ & \cdot {ft}_{a_{i}^{e},a_{i+1}^{e}}^{e}+{\underline{SoC}}^{e}, \end{array}$$

(22) $$at_{a_{i+1}^{e}}^{e}=at_{a_{i}^{e}}^{e}+ft_{a_{i}^{e},a_{i+1}^{e}}^{e}+rw^{e}\cdot ht_{a_{i}^{e}}^{e},\;\;0\leq i<m^{e}$$

(23) $$ft_{a_{i}^{e},a_{i+1}^{e}}^{e}=\frac{∥{\vec{bl}}_{a_{i}^{e},a_{i+1}^{e}}∥}{∥{\vec{av}}_{a_{i}^{e},a_{i+1}^{e}}^{e}∥},\;\;0\leq i<m^{e}$$

where $SoC_{a_{0}^{e}}^{e}$ and ${\underline{SoC}}^{e}$ denote the remaining battery power and the lower limit of battery power at the time of departure of drone *e*, respectively. ${\vec{bl}}_{a_{i}^{e},a_{i+1}^{e}}$ represents the distance vector between the center of the *i*th air-cube $a_{i}^{e}$ and the center of the (*i*+1)th air-cube $a_{i+1}^{e}$ along the route. $RAP^{e}\left(\cdot \right)$ and $FAP^{e}\left(\cdot \right)$ stand for the battery power functions derived from [29,30] for rotary-wing and fixed-wing drones, respectively, and $tlt^{e}$ is the power consumption during takeoff and landing of fixed-wing drones calculated from [30]. ${\vec{av}}_{a_{i}^{e},a_{i+1}^{e}}^{e}$ is the full speed vector of the drone from $a_{i}^{e}$ to $a_{i+1}^{e}$ based on the maximum horizontal and vertical flight speeds of the drone provided by the drone operator, and $ht_{a_{i}^{e}}^{e}$ represents the hovering time of the rotary-wing drone at $a_{i}^{e}$. The values of ${\vec{bl}}_{a_{i}^{e},a_{i+1}^{e}}$, ${\vec{av}}_{a_{i}^{e},a_{i+1}^{e}}^{e}$, and $ht_{a_{i}^{e}}^{e}$ can be derived during the process of flight path calculation by the conflict-free A* algorithm. The binary flag $S_{a_{i}^{e},a_{i+1}^{e}}^{e}\left(at_{a_{i}^{e}}^{e}\right)=1$ represents if the weather is clear at the time $at_{a_{i}^{e}}^{e}$ and if it is suitable for charging when the fixed-wing drone with solar panels flies from $a_{i}^{e}$ to $a_{i+1}^{e}$. $SAP^{e}\left(\cdot \right)$ is the solar charging power function [10], and its parameters $tp_{a_{i}^{e},a_{i+1}^{e}}^{e}\left(at_{a_{i}^{e}}^{e}\right)$ and $si_{a_{i}^{e},a_{i+1}^{e}}^{e}\left(at_{a_{i}^{e}}^{e}\right)$ represent the temperature and solar irradiance at time $at_{a_{i}^{e}}^{e}$, respectively. $\eta^{e}$ is the battery charging efficiency of drone *e*.

Step 3: If the battery of the drone has enough power to reach the destination, proceed to Step 6.

Instead, request the latest traffic and charging information of the air-cubes along the route calculated in Step 1 and that of the surrounding air-cubes close by from the governing local airspace management servers. Then, proceed to the next step to find a suitable charging option to charge the battery of drone *e*.

Step 4: Based on the flight path $R^{e}$ obtained in Step 1, find the nearest $K^{e}$ decentralized laser charging stations and the drones on ordinary missions that can provide power along the route $R^{e}$. The distance measure is computed as follows.

(24) $${dist}_{R^{d},R^{\sigma }}=\frac{∥{\vec{bl}}_{a_{m^{e}}^{e},\alpha_{m^{\sigma }}^{\sigma }}\cdot \left({\vec{bl}}_{a_{0}^{e},a_{m^{e}}^{e}}\times {\vec{bl}}_{\alpha_{0}^{\sigma },\alpha_{m^{\sigma }}^{\sigma }}\right)∥}{∥{\vec{bl}}_{a_{0}^{e},a_{m^{e}}^{e}}\times {\vec{bl}}_{\alpha_{0}^{\sigma },\alpha_{m^{\sigma }}^{\sigma }}∥},$$

(25) $$R^{\sigma }=\left[\left(\alpha_{0}^{\sigma },\alpha_{1}^{\sigma }\right),\left(\alpha_{1}^{\sigma },\alpha_{2}^{\sigma }\right),\cdots ,\left(\alpha_{i}^{\sigma },\alpha_{i+1}^{\sigma }\right),\cdots ,\left(\alpha_{m^{\sigma }-1}^{\sigma },\alpha_{m^{\sigma }}^{\sigma }\right)\right],$$

where ${}^{\sigma }$ is the route of the charging option with the index *σ*. ${\vec{bl}}_{a_{0}^{e},a_{m^{e}}^{e}}$, ${\vec{bl}}_{\alpha_{0}^{\sigma },\alpha_{m^{\sigma }}^{\sigma }}$, and ${\vec{bl}}_{a_{m^{e}}^{e},\alpha_{m^{\sigma }}^{\sigma }}$ represent the straight-line distance vector of drone *e* from the departure point to the destination, the straight-line distance vector from the origin to the destination of charging option *σ*, and the straight-line distance vector from the destination of drone *e* to the destination of charging option *σ*, respectively. 

Step 5: Select the charging points suitable for drone *e* using the following equations.

(26) $$\underset{L^{e}}{\arg\min}\left\{\omega_{1}^{e}\cdot \left({at}_{a_{n^{e}}^{e}}^{e}-{at}_{a_{0}^{e}}^{e}\right)+\omega_{2}^{e}\cdot \sum_{0\leq l\leq L^{e}}\sum_{0\leq i<p_{l,n_{l}^{e}}^{e}}\left\|{\vec{bl}}_{p_{l,i}^{e},p_{l,i+1}^{e}}\right\|+\omega_{3}^{e}\;\;\;\;\;\;\;\;\;\;\;\cdot \sum_{0\leq l<L^{e}}\left[\theta_{p_{l,n_{l}^{e}}^{e}}^{e}\cdot {LCC}_{p_{l,n_{l}^{e}}^{e}}^{e}\left({at}_{p_{l,n_{l}^{e}}^{e}}^{e}\right)\cdot {lcp}_{p_{l,n_{l}^{e}}^{e}}^{e}\cdot {LCT}_{p_{l,n_{l}^{e}}^{e}}^{e}\left({at}_{p_{l,n_{l}^{e}}^{e}}^{e}\right)\;\;\;\;\;\;\;\;\;\;\;+\kappa_{p_{l,n_{l}^{e}}^{e}}^{e}\cdot {DCC}_{p_{l,n_{l}^{e}}^{e}}^{\rho_{l}}\left({at}_{p_{l,n_{l}^{e}}^{e}}^{e}\right)\cdot {dcp}_{p_{l,n_{l}^{e}}^{e}}^{\rho_{l}}\cdot {DCT}_{p_{l,n_{l}^{e}}^{e}}^{\rho_{l}}\left({at}_{p_{l,n_{l}^{e}}^{e}}^{e}\right)\right]\right\},$$

subject to:

(27) $$1\leq L^{e}\leq K^{e}$$

(28) $$F_{l}^{e}=\left[\left(p_{l,0}^{e},p_{l,1}^{e}\right),\left(p_{l,1}^{e},p_{l,2}^{e}\right),\cdots ,\left(p_{l,i}^{e},p_{l,i+1}^{e}\right),\cdots ,\left(p_{l,n_{l}^{e}-1}^{e},p_{l,n_{l}^{e}}^{e}\right)\right],0\leq l\leq L^{e}$$

(29) $$p_{0,0}^{e}=a_{0}^{e},\;p_{L,n_{L}^{e}}^{e}=a_{m^{e}}^{e},$$

(30) $$p_{l,n_{l}^{e}}^{e}=p_{l+1,0}^{e},\;\;0\leq l<L^{e}$$

(31) $$ft_{p_{l,i}^{e},p_{l,i+1}^{e}}^{e}=\frac{∥{\vec{bl}}_{p_{l,i}^{e},p_{l,i+1}^{e}}∥}{∥{\vec{av}}_{p_{l,i}^{e},p_{l,i+1}^{e}}^{e}∥},\;\;0<l<L^{e}$$

(32) $$\theta_{p_{l,n_{l}^{e}}^{e}}^{e}\cdot LCT_{p_{l,n_{l}^{e}}^{e}}^{e}\left(at_{p_{l,n_{l}^{e}}^{e}}^{e}\right)\leq \overset{n_{l+1}^{e}-1}{\underset{i=0}{\sum }}ft_{p_{l+1,i}^{e},p_{l+1,i+1}^{e}}^{e},\;\;0\leq l<L^{e}$$

(33) $$\kappa_{p_{l,n_{l}^{e}}^{e}}^{e}\cdot DCT_{p_{l,n_{l}^{e}}^{e}}^{\rho_{l}}\left(at_{p_{l,n_{l}^{e}}^{e}}^{e}\right)\leq \overset{n_{l+1}^{e}-1}{\underset{i=0}{\sum }}ft_{p_{l+1,i}^{e},p_{l+1,i+1}^{e}}^{e},\;\;0\leq l<L^{e}$$

(34) $$at_{p_{l+1,0}^{e}}^{e}=at_{p_{l,n_{l}^{e}}^{e}}^{e}+\theta_{p_{l,n_{l}^{e}}^{e}}^{e}\cdot LCT_{p_{l,n_{l}^{e}}^{e}}^{e}\left(at_{p_{l,n_{l}^{e}}^{e}}^{e}\right)+\kappa_{p_{l,n_{l}^{e}}^{e}}^{e}\cdot DCT_{p_{l,n_{l}^{e}}^{e}}^{\rho_{l}}\left(at_{p_{l,n_{l}^{e}}^{e}}^{e}\right),0\leq l\leq L^{e}$$

(35) $$SoC_{p_{0,0}^{e}}^{e}>{\underline{SoC}}^{e}+\left(1-rw^{e}\right)\cdot tlt^{e},$$

(36) $${{SoC}_{p_{l,i+1}^{e}}^{e}=SoC}_{p_{l,i}^{e}}^{e}-{rw}^{e}\cdot \left[{RAP}^{e}\left({\vec{av}}_{p_{l,i}^{e},p_{l,i+1}^{e}}^{e}\right)\cdot {ft}_{p_{l,i}^{e},p_{l,i+1}^{e}}^{e}+{{RAP}^{e}\left(0\right)\cdot ht}_{p_{l,i}^{e}}^{e}\right]-\;\left(1-{rw}^{e}\right)\cdot {FAP}^{e}\left({\vec{av}}_{p_{l,i}^{e},p_{l,i+1}^{e}}^{e},{ft}_{p_{l,i}^{e},p_{l,i+1}^{e}}^{e}\right)\cdot {ft}_{p_{l,i}^{e},p_{l,i+1}^{e}}^{e}>\left(1-{rw}^{e}\right)\cdot \eta^{e}\cdot \;S_{p_{l,i}^{e},p_{l,i+1}^{e}}^{e}\left({at}_{p_{l,i}^{e}}^{e}\right)\cdot {SAP}^{e}\left({tp}_{p_{l,i}^{e},p_{l,i+1}^{e}}^{e}\left({at}_{p_{l,i}^{e}}^{e}\right),{si}_{p_{l,i}^{e},p_{l,i+1}^{e}}^{e}\left({at}_{p_{l,i}^{e}}^{e}\right)\right)\cdot {ft}_{p_{l,i}^{e},p_{l,i+1}^{e}}^{e}+\;{\underline{SoC}}^{e},\;\;\;\;\;\;\;0\leq l<L^{e},\;0\leq i<n_{l}^{e}$$

(37) $$\begin{array}{cc} {SoC}_{p_{l+1,0}^{e}}^{e}= & {SoC}_{p_{l,n_{l}^{e}}^{e}}^{e}+\theta_{p_{l,n_{l}^{e}}^{e}}^{e}\cdot \eta^{e}\cdot lcp_{p_{l,n_{l}^{e}}^{e}}^{e}\cdot LCT_{p_{l,n_{l}^{e}}^{e}}^{e}\left(at_{p_{l,n_{l}^{e}}^{e}}^{e}\right)+\kappa_{p_{l,n_{l}^{e}}^{e}}^{e}\cdot \eta^{e}\cdot \\ \; & dcp_{p_{l,n_{l}^{e}}^{e}}^{\rho_{l}}\cdot DCT_{p_{l,n_{l}^{e}}^{e}}^{\rho_{l}}\left(at_{p_{l,n_{l}^{e}}^{e}}^{e}\right)\leq \bar{S_{oC}}{}^{e},0\leq l<L^{e} \end{array}$$

(38) $$at_{p_{l,n_{l}^{e}}^{e}}^{\rho_{l}}\leq at_{p_{l,n_{l}^{e}}^{e}}^{e},if\;\;\kappa_{p_{l,n_{l}^{e}}^{e}}^{e}=1$$

(39) $${\hat{J}}_{\zeta }^{\rho_{l}}=\left[\left({\hat{\alpha }}_{\zeta ,0}^{\rho_{l}},{\hat{\alpha }}_{\zeta ,1}^{\rho_{l}}\right),\left({\hat{\alpha }}_{\zeta ,1}^{\rho_{l}},{\hat{\alpha }}_{\zeta ,2}^{\rho_{l}}\right),\cdots ,\left({\hat{\alpha }}_{\zeta ,i}^{\rho_{l}},{\hat{\alpha }}_{\zeta ,i+1}^{\rho_{l}}\right),\cdots ,\left({\hat{\alpha }}_{\zeta ,{𝓃}_{\zeta }^{\rho_{l}}-1}^{\rho_{l}},{\hat{\alpha }}_{\zeta ,{𝓃}_{\zeta }^{\rho_{l}}}^{\rho_{l}}\right)\right],\;0\leq \zeta \leq 2,\;\;0\leq l<L^{e},if\;\;\kappa_{p_{l,n_{l}^{e}}^{e}}^{e}=1$$

(40) $$J^{\rho_{l}}=\left[\left(\alpha_{0}^{\rho_{l}},\alpha_{1}^{\rho_{l}}\right),\left(\alpha_{1}^{\rho_{l}},\alpha_{2}^{\rho_{l}}\right),\cdots ,\left(\alpha_{i}^{\rho_{l}},\alpha_{i+1}^{\rho_{l}}\right),\cdots ,\left(\alpha_{m^{\rho_{l}}-1}^{\rho_{l}},\alpha_{m^{\rho_{l}}}^{\rho_{l}}\right)\right],0\leq l<L^{e},\;if\;\;\kappa_{p_{l,n_{l}^{e}}^{e}}^{e}=1$$

(41) $${\hat{\alpha }}_{0,0}^{\rho_{l}}=\alpha_{0}^{\rho_{l}},{\hat{\alpha }}_{0,{𝓃}_{0}^{\rho_{l}}}^{\rho_{l}}={\hat{\alpha }}_{1,0}^{\rho_{l}},0\leq l\leq L^{e},if\;\;\kappa_{p_{l,n_{l}^{e}}^{e}}^{e}=1$$

(42) $${\hat{\alpha }}_{1,i}^{\rho_{l}}=p_{l+1,i+1}^{e},0\leq i\leq {𝓃}_{1}^{\rho_{l}},0\leq {𝓃}_{1}^{\rho_{l}}<n_{l+1}^{e},0\leq l<L^{e},if\;\kappa_{p_{l,n_{l}^{e}}^{e}}^{e}=1$$

(43) $${\hat{\alpha }}_{1,{𝓃}_{1}^{\rho_{l}}}^{\rho_{l}}={\hat{\alpha }}_{2,0}^{\rho_{l}}=p_{l+1,{𝓃}_{l+1}^{e}}^{e},{\hat{\alpha }}_{2,{𝓃}_{2}^{\rho_{l}}}^{\rho_{l}}=\alpha_{m^{\rho_{l}}}^{\rho_{l}},0\leq l<L^{e},if\;\kappa_{p_{l,n_{l}^{e}}^{e}}^{e}=1$$

(44) $$at_{{\hat{\alpha }}_{1,0}^{\rho_{l}}}^{\rho_{l}}\leq at_{p_{l,n_{l}^{e}-1}^{e}}^{e},0\leq l<L^{e},if\;\kappa_{p_{l,n_{l}^{e}}^{e}}^{e}=1$$

(45) $${\vec{av}}_{{\hat{\alpha }}_{1,i}^{\rho_{l}},{\hat{\alpha }}_{1,i+1}^{\rho_{l}}}^{\rho_{l}}={\vec{av}}_{p_{l+1,i}^{e},p_{l+1,i+1}^{e}}^{e},\;0\leq i\leq {𝓃}_{1}^{\rho_{l}},0\leq l<L^{e},if\;\kappa_{p_{l,n_{l}^{e}}^{e}}^{e}=1\;$$

(46) $$ee_{p_{l,n_{l}^{e}}^{e}}^{\rho_{l}}=ee_{{\hat{\alpha }}_{0,0}^{\rho_{l}}}^{\rho_{l}}-rw^{\rho_{l}}\cdot \left[\underset{0\leq i<{𝓃}_{0}^{\rho_{l}}}{\sum }RAP^{\rho_{l}}\left({\vec{av}}_{{\hat{\alpha }}_{0,i}^{\rho_{l}},{\hat{\alpha }}_{0,i+1}^{\rho_{l}}}^{\rho_{l}}\right)\cdot ft_{{\hat{\alpha }}_{0,i}^{\rho_{l}},{\hat{\alpha }}_{0,i+1}^{\rho_{l}}}^{\rho_{l}}\right.\;\left.+\underset{0\leq i<{𝓃}_{2}^{\rho_{l}}}{\sum }RAP^{\rho_{l}}\left({\vec{av}}_{{\hat{\alpha }}_{2,i}^{\rho_{l}},{\hat{\alpha }}_{2,i+1}^{\rho_{l}}}^{\rho_{l}}\right)\cdot ft_{{\hat{\alpha }}_{2,i}^{\rho_{l}},{\hat{\alpha }}_{2,i+1}^{\rho_{l}}}^{\rho_{l}}+RAP^{\rho_{l}}\left(0\right)\cdot ht_{{\hat{\alpha }}_{0,0}^{\rho_{l}}}^{\rho_{l}}\right]\;-\left(1-rw^{\rho_{l}}\right)\cdot \left[\underset{0\leq i<{𝓃}_{0}^{\rho_{l}}}{\sum }FAP^{\rho_{l}}\left({\vec{av}}_{{\hat{\alpha }}_{0,i}^{\rho_{l}},{\hat{\alpha }}_{0,i+1}^{\rho_{l}}}^{\rho_{l}},ft_{{\hat{\alpha }}_{0,i}^{\rho_{l}},{\hat{\alpha }}_{0,i+1}^{\rho_{l}}}^{\rho_{l}}\right)\cdot ft_{{\hat{\alpha }}_{0,i}^{\rho_{l}},{\hat{\alpha }}_{0,i+1}^{\rho_{l}}}^{\rho_{l}}\right.+\;\left.\underset{0\leq i<{𝓃}_{2}^{\rho_{l}}}{\sum }FAP^{\rho_{l}}\left({\vec{av}}_{{\hat{\alpha }}_{2,i}^{\rho_{l}},{\hat{\alpha }}_{2,i+1}^{\rho_{l}}}^{\rho_{l}},ft_{{\hat{\alpha }}_{2,i}^{\rho_{l}},{\hat{\alpha }}_{2,i+1}^{\rho_{l}}}^{\rho_{l}}\right)\cdot ft_{{\hat{\alpha }}_{2,i}^{\rho_{l}},{\hat{\alpha }}_{2,i+1}^{\rho_{l}}}^{\rho_{l}}\right]>\;\begin{array}{ll} \left(1-{rw}^{\rho_{l}}\right)\cdot \eta^{\rho_{l}}\cdot & \left[\sum_{0\leq i<{𝓃}_{0}^{\rho_{l}}}S_{{\hat{\alpha }}_{2,i}^{\rho_{l}},{\hat{\alpha }}_{2,i+1}^{\rho_{l}}}^{\rho_{l}}({at}_{{\hat{\alpha }}_{0,i}^{\rho_{l}}}^{\rho_{l}}\right)\\ & \cdot {SAP}^{\rho_{l}}\left({tp}_{{\hat{\alpha }}_{0,i}^{\rho_{l}},{\hat{\alpha }}_{0,i+1}^{\rho_{l}}}^{\rho_{l}}\left({at}_{{\hat{\alpha }}_{0,i}^{\rho_{l}}}^{\rho_{l}}\right),{si}_{{\hat{\alpha }}_{0,i}^{\rho_{l}},{\hat{\alpha }}_{0,i+1}^{\rho_{l}}}^{\rho_{l}}\left({at}_{{\hat{\alpha }}_{0,i}^{\rho_{l}}}^{\rho_{l}}\right)\right)\cdot {ft}_{{\hat{\alpha }}_{0,i}^{\rho_{l}},{\hat{\alpha }}_{0,i+1}^{\rho_{l}}}^{\rho_{l}}\\ & +\sum_{0\leq i<{𝓃}_{2}^{\rho_{l}}}S_{{\hat{\alpha }}_{2,i}^{\rho_{l}},{\hat{\alpha }}_{2,i+1}^{\rho_{l}}}^{\rho_{l}}\left({at}_{{\hat{\alpha }}_{2,i}^{\rho_{l}}}^{\rho_{l}}\right)\\ & \cdot {SAP}^{\rho_{l}}({tp}_{{\hat{\alpha }}_{2,i}^{\rho_{l}},{\hat{\alpha }}_{2,i+1}^{\rho_{l}}}^{\rho_{l}}({at}_{{\hat{\alpha }}_{2,i}^{\rho_{l}}}^{\rho_{l}}),{si}_{{\hat{\alpha }}_{2,i}^{\rho_{l}},{\hat{\alpha }}_{2,i+1}^{\rho_{l}}}^{\rho_{l}}({at}_{{\hat{\alpha }}_{2,i}^{\rho_{l}}}^{\rho_{l}}))\cdot {ft}_{{\hat{\alpha }}_{2,i}^{\rho_{l}},{\hat{\alpha }}_{2,i+1}^{\rho_{l}}}^{\rho_{l}}]\\ & +{\underline{SoC}}^{\rho_{l}}, \end{array}\;0\leq l<L^{e},\;if\;\kappa_{p_{l,n_{l}^{e}}^{e}}^{e}=1$$

(47) $$at_{{\hat{\alpha }}_{j,i+1}^{\rho_{l}}}^{\rho_{l}}=at_{{\hat{\alpha }}_{j,i}^{\rho_{l}}}^{\rho_{l}}+ft_{{\hat{\alpha }}_{j,i}^{\rho_{l}},{\hat{\alpha }}_{j,i+1}^{\rho_{l}}}^{\rho_{l}}+rw^{\rho_{l}}\cdot ht_{{\hat{\alpha }}_{j,i}^{\rho_{l}}}^{\rho_{l}},\;\;j=0,2,\;\;0\leq i<{𝓃}_{j}^{\rho_{l}}$$

(48) $$ft_{{\hat{\alpha }}_{j,i}^{\rho_{l}},{\hat{\alpha }}_{j,i+1}^{\rho_{l}}}^{\rho_{l}}=\frac{∥{\vec{bl}}_{{\hat{\alpha }}_{j,i}^{\rho_{l}},{\hat{\alpha }}_{j,i+1}^{\rho_{l}}}∥}{∥{\vec{av}}_{{\hat{\alpha }}_{j,i}^{\rho_{l}},{\hat{\alpha }}_{j,i+1}^{\rho_{l}}}^{\rho_{l}}∥},\;\;\;\;\;\;j=0,2,\;\;0\leq i<{𝓃}_{j}^{\rho_{l}}$$

(49) $$dcp_{p_{l,n_{l}^{e}}^{e}}^{\rho_{l}}\cdot DCT_{p_{l,n_{l}^{e}}^{e}}^{\rho_{l}}\left(at_{p_{l,n_{l}^{e}}^{e}}^{e}\right)\leq ee_{p_{l,n_{l}^{e}}^{e}}^{\rho_{l}},\;\;0\leq l<L^{e},if\;\kappa_{p_{l,n_{l}^{e}}^{e}}^{e}=1$$

(50) $$\theta_{p_{l,n_{l}^{e}}^{e}}^{e}+\kappa_{p_{l,n_{l}^{e}}^{e}}^{e}\leq 1,\;\;0\leq l<L^{e}$$

(51) $$\underset{0\leq l<L^{e}}{\sum }\left(\theta_{p_{l,n_{l}^{e}}^{e}}^{e}+\kappa_{p_{l,n_{l}^{e}}^{e}}^{e}\right)\geq 1$$

The parameters used in the above equations are defined as follows:

(i) The three parameters from the left to the right in the calculation of arg min(·) represent the flight time of drone *e* from the departure point to the destination, the flight distance, and the charging cost of the of battery of drone *e*. The drone operator can adjust the weight values of $\omega_{1}^{e}$, $\omega_{2}^{e}$, and $\omega_{3}^{e}$ according to the mission characteristics.
(ii) The module allows the drone to charge its battery at multiple charging options, where $F_{0}^{e}$ is the flight path from the departure location to the first charging option calculated by [28], $F_{1}^{e}$ to $F_{L-1}^{e}$ is the flight path from the first charging options through the last charging option determined by [28], and $F_{L}^{e}$ is the flight path from the last charging option to the destination. $at_{a_{0}^{e}}^{e}$ and $at_{a_{n^{e}}^{e}}^{e}$ represent the takeoff time of the drone at the departure point and the arrival time at the destination, respectively. $at_{p_{l,n_{l}^{e}}^{e}}^{e}$ and $at_{p_{l+1,0}^{e}}^{d}$ represent the arrival time of the drone at the *l*th charging option and the takeoff time after the charging is finished at the *l*th charging option, respectively.
(iii) The binary flags $\theta_{p_{l,n_{l}^{e}}^{e}}^{e}$ and $\kappa_{p_{l,n_{l}^{e}}^{e}}^{e}$ indicate whether drone *e* takes use of the distributed laser charging facility located at $p_{l,n_{l}^{e}}^{e}$ or drone-to-drone inflight wireless charging, respectively. The binary flag for the selected charging option will be set to one, otherwise it will be zero. ${\bar{SoC}}^{e}$ represents the upper limit of the battery capacity, $SoC_{p_{l,n_{l}^{e}}^{e}}^{e}$ stands for the remaining battery capacity of the drone at $p_{l,n_{l}^{e}}^{e}$, and $SoC_{p_{l+1,0}^{e}}^{e}$ denotes the battery capacity of drone *e* after charging at $p_{l,n_{l}^{e}}^{e}$.
(iv) $\rho_{l}$ is the index value for the drone providing power at $p_{l,n_{l}^{e}}^{e}$, and $\eta^{\rho_{l}}$ is the battery charging efficiency of drone $\rho_{l}$. $LCC_{p_{l,n_{l}^{e}}^{e}}^{e}\left(at_{p_{l,n_{l}^{e}}^{e}}^{e}\right)$ and $DCC_{p_{l,n_{l}^{e}}^{e}}^{\rho_{l}}\left(at_{p_{l,n_{l}^{e}}^{e}}^{e}\right)$ denote the real-time charging price of decentralized laser charging facilities at $p_{l,n_{l}^{e}}^{e}$ and that of drone *ρ_l_* at time $at_{p_{l,n_{l}^{e}}^{e}}^{e}$, respectively. $lcp_{p_{l,n_{l}^{e}}^{e}}^{e}$ and $dcp_{p_{l,n_{l}^{e}}^{e}}^{\rho_{l}}$ represent the charging power of the decentralized laser charging facility and that of drone $\rho_{l}$ at $p_{l,n_{l}^{e}}^{e}$, respectively. $LCT_{p_{l,n_{l}^{e}}^{e}}^{e}\left(at_{p_{l,n_{l}^{e}}^{e}}^{e}\right)$ and $DCT_{p_{l,n_{l}^{e}}^{e}}^{\rho_{l}}\left(at_{p_{l,n_{l}^{e}}^{e}}^{e}\right)$ denote the actual charging time of drone e’s battery via the distributed laser charging facility and that via drone-to-drone wireless charging at $p_{l,n_{l}^{e}}^{e}$, respectively.
(v) The binary flags $S_{p_{l,i}^{e},p_{l,i+1}^{e}}^{e}$(·), $S_{{\hat{\alpha }}_{0,i}^{\rho_{l}},{\hat{\alpha }}_{0,i+1}^{\rho_{l}}}^{\rho_{l}}\left(\cdot \right)$, and $S_{{\hat{\alpha }}_{2,i}^{\rho_{l}},{\hat{\alpha }}_{2,i+1}^{\rho_{l}}}^{\rho_{l}}\left(\cdot \right)$ indicate whether the fixed-wing drone is suitable for solar power charging; $tp_{p_{l,i}^{e},p_{l,i+1}^{e}}^{e}\left(\cdot \right)$, $tp_{{\hat{\alpha }}_{0,i}^{\rho_{l}},{\hat{\alpha }}_{0,i+1}^{\rho_{l}}}^{\rho_{l}}\left(\cdot \right)$, and $tp_{{\hat{\alpha }}_{2,i}^{\rho_{l}},{\hat{\alpha }}_{2,i+1}^{\rho_{l}}}^{\rho_{l}}\left(\cdot \right)$ is air temperature, while $si_{p_{l,i}^{e},p_{l,i+1}^{e}}^{e}\left(\cdot \right)$, $si_{{\hat{\alpha }}_{0,i}^{\rho_{l}},{\hat{\alpha }}_{0,i+1}^{\rho_{l}}}^{\rho_{l}}\left(\cdot \right)$, and $si_{{\hat{\alpha }}_{2,i}^{\rho_{l}},{\hat{\alpha }}_{2,i+1}^{\rho_{l}}}^{\rho_{l}}\left(\cdot \right)$ represents the solar irradiance.
(vi) $J^{\rho_{l}}$ represents the flight path before drone $\rho_{l}$ changes its route, and ${\hat{J}}_{1}^{\rho_{l}}$ is the flight path that is used by drone $\rho_{l}$ to provide power to drone *e* through drone-to-drone charging. ${\hat{J}}_{0}^{\rho_{l}}$ and ${\hat{J}}_{2}^{\rho_{l}}$ denote the flight path that drone $\rho_{l}$ flies to the rendezvous point and the flight path that drone $\rho_{l}$ flies back to the original planned route after discharging, respectively. Notably, the flight path ${\hat{J}}_{1}^{\rho_{l}}$ of drone $\rho_{l}$ and $F_{l+1}^{e}$ of drone *e* maintain a distance of one air-cube during drone-to-drone wireless charging. In addition, the arrival time of drone $\rho_{l}$ at the starting point of ${\hat{J}}_{1}^{\rho_{l}}$ must be earlier than the arrival time of drone *e* at the starting point of $F_{l+1}^{e}$ to avoid delaying the scheduled trip of drone *e*. Meanwhile, the flight speed ${\vec{av}}_{{\hat{\alpha }}_{1,i}^{\rho_{l}},{\hat{\alpha }}_{1,i+1}^{\rho_{l}}}^{\rho_{l}}$ of drone $\rho_{l}$ must also be identical to the flight speed ${\vec{av}}_{p_{l+1,i}^{e},p_{l+1,i+1}^{e}}^{e}$ *o*f drone *e* during drone-to-drone charging.
(vii) $ee_{{\hat{\alpha }}_{0,0}^{\rho_{l}}}^{\rho_{l}}$ and $ee_{p_{l,n_{l}^{e}}^{e}}^{\rho_{l}}$ represent the battery power of drone $\rho_{l}$ before it changes its route and the battery power right before it starts drone-to-drone wireless charging. Since drone $\rho_{l}$ needs to fly two additional flight segments ${\hat{J}}_{0}^{\rho_{l}}$ and ${\hat{J}}_{2}^{\rho_{l}}$ to charge the battery of drone *e*, the battery power reaching the rendezvous point $ee_{p_{l,n_{l}^{e}}^{e}}^{\rho_{l}}$ should be deducted from the battery power consumed by the two additional flight segments. The latest information of $ee_{{\hat{\alpha }}_{0,0}^{\rho_{l}}}^{\rho_{l}}$ is provided by the governing local airspace management server.

Step 6: After determining the flight route and flight time from the departure point to the charging point and from the charging point to the destination, the flight and charging-related information of drone *e* is transmitted to the local segment management server.

Step 7: If any drones on ordinary missions yield airspace for drone use, this module will activate the module in the subsequent subsection to adjust the flight paths of the drones that yield the air-cubes immediately.

### 2.3. Real-Time Flight Route and Charging Planning for a Drone on an Ordinary Mission

Due to weather conditions, a drone may not arrive at the designated air-cubes along its route on time. The drone on a time-critical mission may occupy the air-cubes originally planned for a drone on an ordinary mission. In both cases, this module will be activated to adjust the drones’ flight paths. When pre-planning the flight path before takeoff, the drones fly at full speed and maintain a low altitude to save power and shorten the flight time [28]. Since there is no time constraint for a drone on an ordinary mission to complete its mission, this module will attempt to maintain the original or similar flight paths as much as possible. To be specific, a fixed-wing drone can reduce its flight speed, whereas a rotary-wing drone can choose to reduce the speed, extend the drones’ hovering time in the air-cubes it travels through, or increase its flight altitude.

Assume that a drone on an ordinary mission indexed by *d* originally planned a flight path of $R^{d}$ as follows.

(52) $$R^{d}=\left[\left(a_{0}^{d},a_{1}^{d}\right),\left(a_{1}^{d},a_{2}^{d}\right),\cdots ,\left(a_{i}^{d},a_{i+1}^{d}\right),\cdots ,\left(a_{\cdot^{d}-1}^{d},a_{\cdot^{d}}^{d}\right)\right],$$

The detailed steps of adjusting the drone’s flight path are given below.

Step 1: The flight path of drone *d* is corrected based on the following equations:

(53) $$\underset{{\hat{m}}^{d}}{\arg\min}\;\left\{{𝓌}_{1}^{d}\cdot \left(\underset{0\leq i<{\hat{m}}^{d}}{\sum }∥{\vec{bl}}_{{\hat{a}}_{i}^{d},{\hat{a}}_{i+1}^{d}}∥-\underset{0\leq i<{\hat{m}}^{d}}{\sum }∥{\vec{bl}}_{{\hat{a}}_{i}^{d},{\hat{a}}_{i+1}^{d}}∥\right)+{𝓌}_{2}^{d}\;\cdot \left(at_{{\hat{a}}_{{\hat{\cdot }}^{d}}^{d}}^{d}-at_{a_{0}^{d}}^{d}\right)\right\}.\;$$

subject to:

(54) $${\hat{R}}^{d}=\left[\left({\hat{a}}_{0}^{d},{\hat{a}}_{1}^{d}\right),\left({\hat{a}}_{1}^{d},{\hat{a}}_{2}^{d}\right),\cdots ,\left({\hat{a}}_{i}^{d},{\hat{a}}_{i+1}^{d}\right),\cdots ,\left({\hat{a}}_{{\hat{m}}^{d}-1}^{d},{\hat{a}}_{{\hat{m}}^{d}}^{d}\right)\right]$$

(55) $${\hat{a}}_{0}^{d}=a_{0}^{d}$$

(56) $${\underline{\vec{av}}}^{d}\leq {\vec{nav}}_{{\hat{a}}_{i}^{d},{\hat{a}}_{i+1}^{d}}^{d}=\varepsilon_{{\hat{a}}_{i}^{d},{\hat{a}}_{i+1}^{d}}^{d}\cdot {\vec{av}}_{{\hat{a}}_{i}^{d},{\hat{a}}_{i+1}^{d}}^{d},\;0\leq \varepsilon_{{\hat{a}}_{i}^{d},{\hat{a}}_{i+1}^{d}}^{d}\leq 1,\;0\leq i<{\hat{m}}^{d}$$

(57) $$nft_{{\hat{a}}_{i}^{d},{\hat{a}}_{i+1}^{d}}^{d}=\frac{∥{\vec{bl}}_{{\hat{a}}_{i}^{d},{\hat{a}}_{i+1}^{d}}∥}{∥{\vec{nav}}_{{\hat{a}}_{i}^{d},{\hat{a}}_{i+1}^{d}}^{d}∥},\;\;\;0\leq i<{\hat{m}}^{d}$$

(58) $${{SoC}_{{\hat{a}}_{i+1}^{d}}^{d}=SoC}_{{\hat{a}}_{i}^{d}}^{d}-{rw}^{d}\cdot \left[{RAP}^{d}\left({\vec{nav}}_{{\hat{a}}_{i}^{d},{\hat{a}}_{i+1}^{d}}^{d}\right)\cdot {nft}_{{\hat{a}}_{i}^{d},{\hat{a}}_{i+1}^{d}}^{d}+{RAP}^{d}\left(0\right)\cdot \;\left({{ht}_{{\hat{a}}_{i}^{d}}^{d}+nht}_{{\hat{a}}_{i}^{d}}^{d}\right)\right]-\left(1-{rw}^{d}\right)\cdot {FAP}^{d}\left({\vec{nav}}_{{\hat{a}}_{i}^{d},{\hat{a}}_{i+1}^{d}}^{d},{nft}_{{\hat{a}}_{i}^{d},{\hat{a}}_{i+1}^{d}}^{d}\right)\cdot {nft}_{{\hat{a}}_{i}^{d},{\hat{a}}_{i+1}^{d}}^{d}>\;\left(1-{rw}^{d}\right)\cdot \eta^{d}\cdot S_{{\hat{a}}_{i}^{d},{\hat{a}}_{i+1}^{d}}^{d}\left({at}_{{\hat{a}}_{i}^{d}}^{d}\right)\cdot {SAP}^{d}\left({tp}_{{\hat{a}}_{i}^{d},{\hat{a}}_{i+1}^{d}}^{d}\left({at}_{{\hat{a}}_{i}^{d}}^{d}\right),{si}_{{\hat{a}}_{i}^{d},{\hat{a}}_{i+1}^{d}}^{d}\left({at}_{{\hat{a}}_{i}^{d}}^{d}\right)\right)\cdot \;{nft}_{{\hat{a}}_{i}^{d},{\hat{a}}_{i+1}^{d}}^{d}+{\underline{SoC}}^{d},\;\;\;\;0\leq i<{\hat{m}}^{d}$$

(59) $$\phi_{{\hat{a}}_{{\hat{m}}^{d}}^{d}}^{d}\cdot \left(\eta^{d}\cdot {pcp}_{{\hat{a}}_{{\hat{m}}^{d}}^{d}}^{d}\cdot {ct}_{{\hat{a}}_{{\hat{m}}^{d}}^{d}}^{d}+{SoC}_{{\hat{a}}_{{\hat{m}}^{d}}^{d}}^{d}\right)\leq {\bar{SoC}}^{d},\;$$

(60) $$\phi_{{\hat{a}}_{{\hat{m}}^{d}}^{d}}^{d}+\Psi_{{\hat{a}}_{{\hat{m}}^{d}}^{d}}^{d}\leq 1,\;$$

(61) $$ee_{{\hat{a}}_{i}^{d}}^{d}={SoC}_{{\hat{a}}_{{\hat{m}}^{d}}^{d}}^{d}-{\underline{SoC}}^{d},\;\;0\leq i<{\hat{m}}^{d}$$

The parameters used in the above equations are defined as follows:

(i) The first parameter used in the calculation of arg min(·) represents the difference between the flight length of the new route and the original route of drone *d* due to the route correction. The second parameter denotes the flight time of drone *d* from the departure point to the destination, respectively. Drone operators can flexibly adjust the weighting of ${𝓌}_{1}^{d}$ and ${𝓌}_{2}^{d}$ according to mission characteristics and operating cost considerations.
(ii) ${\hat{R}}^{d}$ is the adjusted flight path by using the conflict-free A* algorithm [28]. Since the new route may need to avoid other moving drones and pass through the surrounding air-cubes close to the original route, the index value ${\hat{a}}_{i}^{d}$ is assigned to the *i*th air-cube on the new route other than the starting point $a_{0}^{d}$, and the last air-cube on the route is ${\hat{a}}_{{\hat{m}}^{d}}^{d}$. Since the new route may have insufficient power, the module will also check the battery capacity of drone *d* and direct it to the nearest charging point to recharge the batteries if needed.
(iii) ${\vec{nav}}_{{\hat{a}}_{i}^{d},{\hat{a}}_{i+1}^{d}}^{d}$ is the original full flight speed of drone *d* from ${\hat{a}}_{i}^{d}$ to ${\hat{a}}_{i+1}^{d}$, and ${\vec{av}}_{{\hat{a}}_{i}^{d},{\hat{a}}_{i+1}^{d}}^{d}$ is the speed of drone *d* after deceleration. $\varepsilon_{{\hat{a}}_{i}^{d},{\hat{a}}_{i+1}^{d}}^{d}$ is used to adjust the flight speed of the drone, and $nft_{{\hat{a}}_{i}^{d},{\hat{a}}_{i+1}^{d}}^{d}$ is the time taken by drone *d* to fly from ${\hat{a}}_{i}^{d}$ to ${\hat{a}}_{i+1}^{d}$ after adjusting its speed. $nht_{{\hat{a}}_{i}^{d}}^{d}$ is the possible additional hovering time of the drone at ${\hat{a}}_{i}^{d}$. The other parameters are defined in the same way as those used in Section 2.1.

Step 2: After determining the flight route and flight time from the departure point to the charging point and from the charging point to the destination, the flight and charging-related information is submitted to the governing local airspace management servers.

### 2.4. Real-Time Flight Route and Charging Planning for a Drone on a Time-Critical Mission

This module is used to adjust the flight route of a drone on a time-critical mission if it is unable to reach the designated air-cubes on time due to weather conditions. The drone on a time-critical mission can prioritize the use of air-cubes that have been planned to be passed through by the drones on ordinary missions. However, if the updated air-cubes that the drone will pass through collide with other drones on time-critical missions, the difference between the established mission deadline and the updated arrival time at the destination is adopted as the criterion to determine the drone that can use the collided air-cubes. The flight paths of drones that yield the air-cubes will also be adjusted in this module.

Assume that the flight path of a drone on a time-critical mission that requires a route correction due to weather conditions, $R^{e}$, is given below.

(62) $$R^{e}=\left[F_{0}^{e},\cdots ,F_{l}^{e},\cdots ,F_{L^{e}}^{e}\right],\;0\leq l\leq L^{e}$$

(63) $$F_{l}^{e}=\left[\left(p_{l,0}^{e},p_{l,1}^{e}\right),\left(p_{l,1}^{e},p_{l,2}^{e}\right),\cdots ,\left(p_{l,i}^{e},p_{l,i+1}^{e}\right),\cdots ,\left(p_{l,n_{l}^{e}-1}^{e},p_{l,n_{l}^{e}}^{e}\right)\right],\;0\leq l\leq L^{e}$$

(64) $${\hat{at}}_{p_{l,i}^{e}}^{e}=at_{p_{l,i}^{e}}^{e}+\Delta^{e},\;0\leq i\leq n_{l}^{e},\;0\leq l\leq L^{e}$$

where *e* is the index of the drone on a time-critical mission, and $at_{p_{l,i}^{e}}^{e}$ and ${\hat{at}}_{p_{l,i}^{e}}^{e}$ represent the arrival time of drone *e* at air-cube $p_{l,i}^{e}$ before and after the update of its current route, respectively. Δ^*e*^ is the time difference of the two routes. When $\Delta^{e}$ is negative, it implies that the drone arrives at the air-cube $p_{l,i}^{e}$ earlier than the original time $at_{p_{l,i}^{e}}^{e}$. $L^{e}$ stands for the number of charging facilities for charging drone *e* on the way to its destination.

The detailed steps of this module are given below.

Step 1: Based on the flight information of drones, identify the drones on time-critical missions with overlapping air-cubes on the routes to their destinations as follows.

(65) $$R^{c}=\left[F_{0}^{c},\cdots ,F_{i}^{c},\cdots ,F_{L^{e}}^{e}\right],\;0\leq l\leq L^{c}$$

(66) $$F_{l}^{c}=\left[\left(p_{𝓁,0}^{c},p_{𝓁,1}^{c}\right),\left(p_{𝓁,2}^{c},p_{𝓁,3}^{c}\right),\cdots ,\left(p_{𝓁,j}^{c},p_{𝓁,j+1}^{c}\right),\cdots ,\left(p_{𝓁,n_{𝓁}^{c}-1}^{c},p_{𝓁,n_{𝓁}^{c}}^{c}\right)\right],\;0\leq 𝓁\leq L^{c}$$

(67) $${\hat{at}}_{p_{l,i}^{e}}^{e}-\frac{ft_{p_{l,i-1}^{e},p_{l,i}^{e}}^{e}}{2}\leq {\hat{at}}_{p_{𝓁,j}^{c}}^{c}\leq {\hat{at}}_{p_{l,i}^{e}}^{e}+\frac{ft_{p_{l,i}^{e},p_{l,i+1}^{e}}^{e}}{2},\;\;\;\;\;\;\;\;\;\;\;\;\;\;0\leq i\leq n_{l}^{e},\;0\leq l\leq L^{e},0\leq j\leq n_{𝓁}^{c},\;0\leq 𝓁\leq L^{c}$$

(68) $$ft_{p_{l,i}^{e},p_{l,i+1}^{e}}^{e}=\frac{∥{\vec{bl}}_{p_{l,i}^{e},p_{l,i+1}^{e}}∥}{∥{\vec{av}}_{p_{l,i}^{e},p_{l,i+1}^{e}}^{e}∥},\;0\leq i\leq n_{l}^{e},\;0\leq l\leq L^{e}$$

(69) $$p_{𝓁,j}^{c}=p_{l,i}^{e},0\leq i\leq n_{l}^{e},\;0\leq l\leq L^{e},0\leq j\leq n_{𝓁}^{c},\;0\leq 𝓁\leq L^{c}$$

where $R^{c}$ is the flight path of drone *c* whose air-cubes collide with drone *e*. $L^{c}$ represents the number of charging facilities for charging drone *c* on the way to its destination. ${\hat{at}}_{p_{𝓁,j}^{c}}^{c}$ and ${\hat{at}}_{p_{l,i}^{e}}^{e}$ stand for the arrival time of drone *c* and that of drone *e* at air-cube $p_{l,i}^{e}$, respectively. $ft_{p_{l,i}^{e},p_{l,i+1}^{e}}^{e}$ is the flight time from the center of $p_{l,i}^{e}$ to that of $p_{l,i+1}^{e}$.

Step 2: The flight paths of all drones that yield their air-cubes are determined by,

(70) $$\min\left[w_{1}\cdot \underset{1\leq c\leq C}{\sum }\left({\hat{at}}_{p_{L^{c},n_{L^{c}}^{c}}^{c}}^{c}-dl_{p_{L^{c},n_{L^{c}}^{c}}^{c}}^{c}\right)+w_{2}\cdot \underset{1\leq c\leq C}{\sum }\left({\hat{at}}_{p_{L^{c},n_{L^{c}}^{c}}^{c}}^{c}-{\hat{at}}_{p_{0,0}^{c}}^{c}\right)\right]$$

subject to:

(71) $${\underline{\vec{av}}}^{c}\leq {\vec{nav}}_{p_{𝓁,j}^{c},p_{𝓁,j+1}^{c}}^{c}=\varepsilon_{p_{𝓁,j}^{c},p_{𝓁,j+1}^{c}}^{c}\cdot {\vec{av}}_{p_{𝓁,j}^{c},p_{𝓁,j+1}^{c}}^{c},0\leq \varepsilon_{p_{𝓁,j}^{c},p_{𝓁,j+1}^{c}}^{c}\leq 1,0\leq j\leq n_{𝓁}^{c},0\leq 𝓁\leq L^{c}$$

(72) $$nft_{p_{𝓁,j}^{c},p_{𝓁,j+1}^{c}}^{c}=\frac{∥{\vec{bl}}_{p_{𝓁,j}^{c},p_{𝓁,j+1}^{c}}∥}{∥{\vec{nav}}_{p_{𝓁,j}^{c},p_{𝓁,j+1}^{c}}^{c}∥},\;\;\;0\leq j\leq n_{𝓁}^{c},\;0\leq 𝓁\leq L^{c}$$

(73) $$\theta_{p_{𝓁,n_{𝓁}^{c}}^{c}}^{c}\cdot {\mathrm{LCT}}_{p_{𝓁,n_{𝓁}^{c}}^{c}}^{c}\left({\hat{at}}_{p_{𝓁,n_{𝓁}^{c}}^{c}}^{c}\right)\overset{n_{𝓁+1}^{c}-1}{\underset{j=0}{\sum }}nft_{p_{𝓁+1,j}^{c},p_{𝓁+1,j+1}^{c}}^{c},\;\;\;0\leq 𝓁\leq L^{c}$$

(74) $$\kappa_{p_{𝓁,n_{𝓁}^{c}}^{c}}^{c}\cdot {\mathrm{DCT}}_{p_{𝓁,n_{𝓁}^{c}}^{c}}^{\rho_{𝓁}}\left({\hat{at}}_{p_{𝓁,n_{𝓁}^{c}}^{c}}^{c}\right)\overset{n_{𝓁+1}^{c}-1}{\underset{j=0}{\sum }}nft_{p_{𝓁+1,j}^{c},p_{𝓁+1,j+1}^{c}}^{c},\;\;\;0\leq 𝓁\leq L^{c}$$

(75) $${{SoC}_{p_{𝓁,j+1}^{c}}^{c}=SoC}_{p_{𝓁,j}^{c}}^{c}-{rw}^{c}\cdot \left[{RAP}^{c}\left({\vec{nav}}_{p_{𝓁,j}^{c},p_{𝓁,j+1}^{c}}^{c}\right)\cdot {nft}_{p_{𝓁,j}^{c},p_{𝓁,j+1}^{c}}^{c}+{RAP}^{c}\left(0\right)\cdot \;\left({{ht}_{p_{𝓁,j}^{c}}^{c}+nht}_{p_{𝓁,j}^{c}}^{c}\right)\right]-\left(1-{rw}^{c}\right)\cdot {FAP}^{c}\left({\vec{nav}}_{p_{𝓁,j}^{c},p_{𝓁,j+1}^{c}}^{c},{nft}_{p_{𝓁,j}^{c},p_{𝓁,j+1}^{c}}^{c}\right)\cdot \;{nft}_{p_{𝓁,j}^{c},p_{𝓁,j+1}^{c}}^{c}>\left(1-{rw}^{c}\right)\cdot \eta^{c}\cdot S_{p_{𝓁,j}^{c},p_{𝓁,j+1}^{c}}^{c}\left({at}_{p_{𝓁,j}^{c}}^{c}\right)\cdot \;{SAP}^{c}\left({tp}_{p_{𝓁,j}^{c},p_{𝓁,j+1}^{c}}^{c}\left({at}_{p_{𝓁,j}^{c}}^{c}\right),{si}_{p_{𝓁,j}^{c},p_{𝓁,j+1}^{c}}^{c}\left({at}_{p_{𝓁,j}^{c}}^{c}\right)\right)\cdot {nft}_{p_{𝓁,j}^{c},p_{𝓁,j+1}^{c}}^{c}+{\underline{SoC}}^{c},\;0\leq j\leq \;n_{𝓁}^{c},\;0\leq 𝓁\leq L^{c}$$

(76) $$\theta_{p_{𝓁,n_{𝓁}^{c}}^{c}}^{c}+\kappa_{p_{𝓁,n_{𝓁}^{c}}^{c}}^{c}\leq 1,\;0\leq l<L^{c},\;if\;L^{c}>0$$

(77) $${\hat{at}}_{p_{𝓁+1,0}^{c}}^{c}={\hat{at}}_{p_{𝓁,n_{𝓁}^{c}}^{c}}^{c}+\theta_{p_{𝓁,n_{𝓁}^{c}}^{c}}^{c}\cdot {\mathrm{LCT}}_{p_{𝓁,n_{𝓁}^{c}}^{c}}^{c}\left({\hat{at}}_{p_{𝓁,n_{𝓁}^{c}}^{c}}^{c}\right)+\kappa_{p_{𝓁,n_{𝓁}^{c}}^{c}}^{c}\cdot {\mathrm{DCT}}_{p_{𝓁,n_{𝓁}^{c}}^{c}}^{\rho_{𝓁}}\left({\hat{at}}_{p_{𝓁,n_{𝓁}^{c}}^{c}}^{c}\right),\;0\leq 𝓁\leq L^{c}$$

(78) $${\vec{nav}}_{{\hat{\alpha }}_{1,i}^{\rho_{l}},{\hat{\alpha }}_{1,i+1}^{\rho_{l}}}^{\rho_{l}}={\vec{nav}}_{p_{𝓁+1,j}^{c},p_{𝓁+1,j+1}^{c}}^{c},\;0\leq i\leq {𝓃}_{1}^{\rho_{l}},\;0\leq 𝓁<L^{c},\;if\;\kappa_{p_{𝓁,n_{𝓁}^{c}}^{c}}^{c}=1$$

(79) $$dcp_{p_{𝓁,n_{𝓁}^{c}}^{c}}^{\rho_{l}}\cdot {\text{DCT}}_{p_{𝓁,n_{𝓁}^{c}}^{c}}^{\rho_{l}}\left({\hat{at}}_{p_{𝓁,n_{𝓁}^{c}}^{c}}^{c}\right)\leq ee_{p_{𝓁,n_{𝓁}^{c}}^{c}}^{\rho_{l}},\;0\leq 𝓁<L^{c},\;if\;\kappa_{p_{𝓁,n_{𝓁}^{c}}^{c}}^{c}=1$$

(80) $$\underset{0\leq 𝓁<L^{c}}{\sum }\left(\theta_{p_{𝓁,n_{𝓁}^{c}}^{c}}^{c}+\kappa_{p_{𝓁,n_{𝓁}^{c}}^{c}}^{c}\right)\geq 1,\;\mathrm{if}\;L^{c}>0$$

(81) $${\hat{at}}_{{\hat{\alpha }}_{1,0}^{\rho_{l}}}^{\rho_{l}}={\hat{at}}_{{\hat{\alpha }}_{1,0}^{\rho_{l}}}^{\rho_{l}}+{\hat{at}}_{p_{𝓁+1,0}^{c}}^{c}-at_{p_{𝓁+1,0}^{c}}^{c},\;0\leq 𝓁<L^{c},\;\mathrm{if}\;\kappa_{p_{𝓁,n_{𝓁}^{c}}^{c}}^{c}=1$$

The parameters used in the above equations are defined as follows:

(i) C is the number of drones that yield their air-cubes, ${\hat{R}}^{c}$ denotes the flight path of drone *c*, and ${\hat{at}}_{a_{{\hat{m}}^{d}}^{c}}^{c}$ and $dl_{a_{{\hat{m}}^{d}}^{c}}^{c}$ represent the arrival time of the drone *c* at its destination and the expected deadline for completing the mission, respectively.
(ii) The two parameters in the calculation of min(·) represent the deadline for a drone with overlapping airspace to complete its mission as close as possible to the individually established deadline after route correction and the flight time required for drone *c* to reach its destination from the departure point, respectively. Drone operators can flexibly adjust the weighting of $w_{1}$ and $w_{2}$ according to the urgent mission requirements.
(iii) $\rho_{l}$ is the drone that provides power to drone *c*. The other parameters are defined in the same way as in Section 2.3.

Step 3: If any drones on time-critical missions cannot comply with restrictions specified in Step 2, the module stated in Section 2.2 is activated to modify the flight routes of the corresponding drones.

Step 4: If any of the drones that provide power are not able to comply with restrictions specified in Step 2, the module stated in Section 2.1 is activated to correct the flight path of the corresponding drones.

## 3. Experimental Results and Discussion

**Figure 3:**
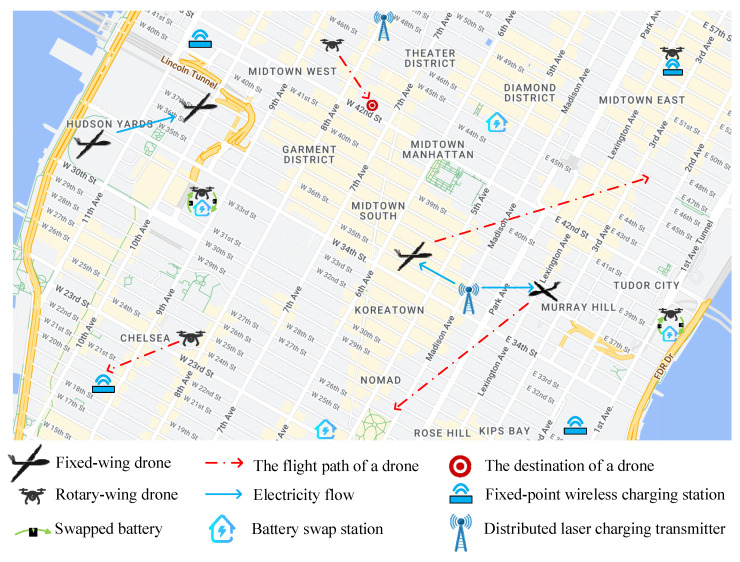
Illustration of a partial simulation scenario.

This study ran a series of simulations to examine the effectiveness of the proposed algorithm. The simulations were performed on a PC with Intel Core i7 at 2.9 GHz CPU and 64 GB RAM. Single-day orders were generated by taking into account the time periods of consumers’ online shopping habits [31] and the inclusion of drone-deliverable goods and items, including traditional ordinary cargo orders and time-critical goods deliveries, such as medical and pharmaceutical products, meals, and fresh food. The requests for cargo orders were created at the corresponding time periods during a day with the aforementioned goods order generation rate. The departure locations and the destinations of all drones were randomly selected within a 625-km square region, which is approximately the scale of a metropolitan-area such as New York City. Figure 3 illustrates a small portion of the simulation scenario. In our simulation, 20 wireless charging stations, 20 battery swap stations, and 10 distributed laser charging services for drones were evenly distributed in the simulation area for charging drones. The total number of the missions was 60,675, including 42,765 ordinary cargo delivery orders and 17,910 time-critical missions. When the simulation was started, the time, distance, start time, and final completion time of each mission were recorded. The time of charging request, the time of starting charging, the time of arriving at the charging point, the time of finishing charging, and the amount of electricity obtained after charging were also recorded when a drone needed to charge while performing its mission.

**Figure 4:**
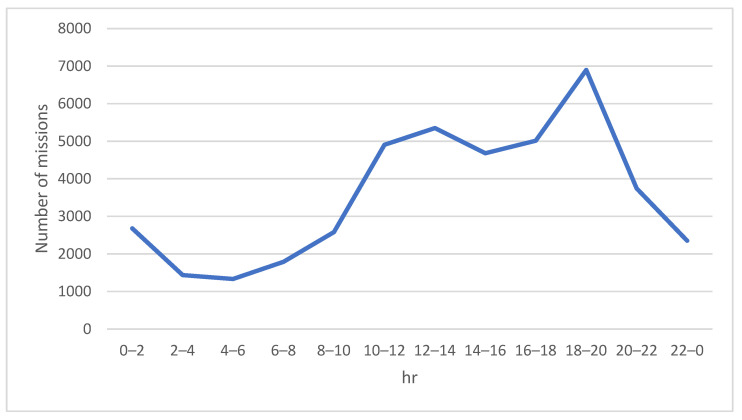
Volume of ordinary missions performed by the drones within a day.

**Figure 5:**
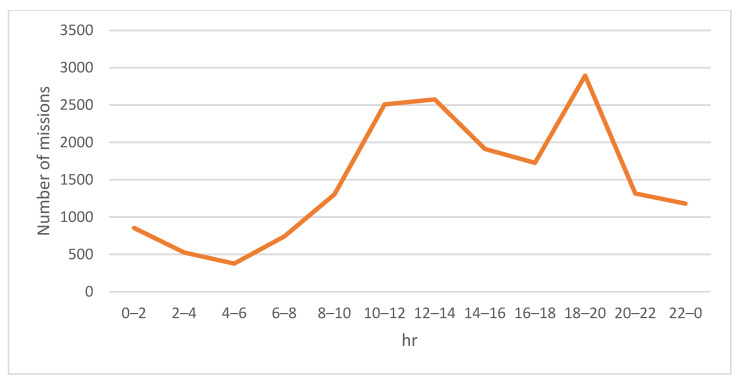
Volume of time-critical missions performed by the drones within a day.

Figure 4 and Figure 5 show the number of ordinary missions and that of time-critical missions, respectively. A small number of orders for goods was observed between midnight and morning peak hours. From around 08:00 onwards, the frequency of consumer orders gradually rose until the lunch break ended. Evening was the peak period when consumers placed the highest frequency of delivering goods orders. After the evening peak period, the number of orders decreased sharply before midnight. 

**Figure 6:**
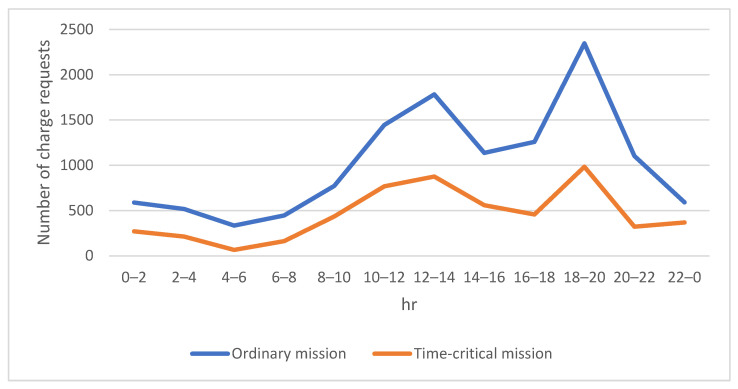
Number of charging requests for drones within a day.

Figure 6 shows the number of charging requests for drones within a day. It can be seen that the counts of charging requests were directly proportional to the number of missions. Accordingly, the charging requests increased significantly during morning and evening peak hours because the drones consumed a lot of electricity while delivering goods during busy periods.

**Figure 7:**
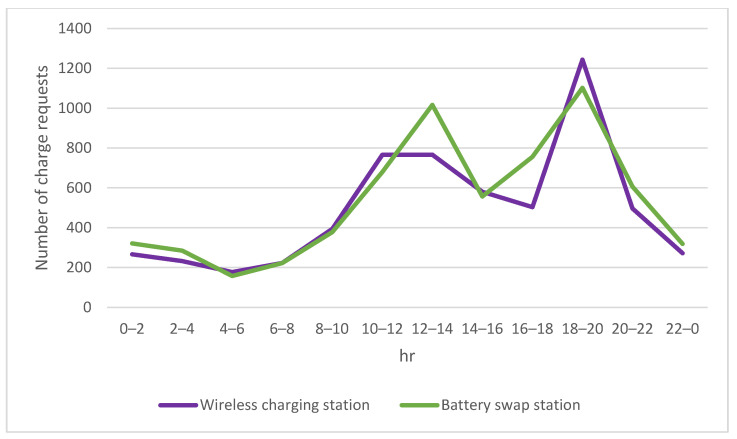
Number of charging requests for ordinary missions.

As there is no pressure on the delivery time for ordinary delivery orders carried by the drones, the preferred charging options for this type of mission will be fixed-point wireless charging stations or battery swap stations due to the operation cost consideration as shown in Figure 7. The number of charging requests surged during morning and evening peak periods as expected.

**Figure 8:**
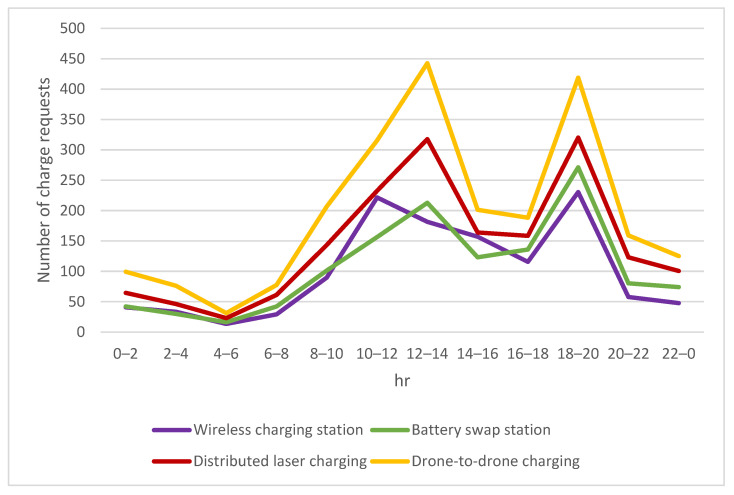
Number of charging requests for time-critical missions.

Since time-critical orders need to be delivered to the destination before the deadline designated by the consumers, the primary charging option is either distributed laser charging facilities or drone-to-drone charging for this type of delivery mission. As observed from Figure 8, it is clear that the use of distributed laser charging facilities and drone-to-drone charging for time-critical orders was much higher than the use of other charging options, including wireless charging stations and battery swap stations. Only when the desired distributed laser charging and drone-to-drone charging were not available, the traditional fixed-point wireless charging or battery swap stations will be chosen for charging.

**Figure 9:**
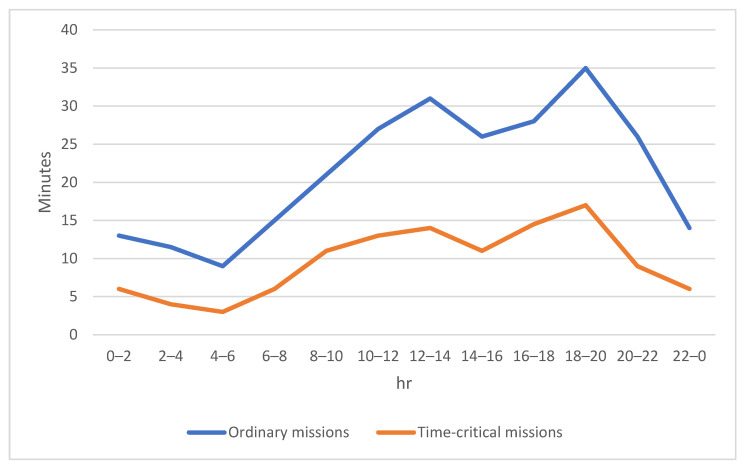
Extra time for a drone spent on charging within a day.

When a drone on the time-critical mission adopted the distributed laser charging option, it always kept a fixed distance between the drone and the designated distributed laser charging facility during in-flight charging. If a drone-to-drone charging option was chosen, both drones kept a fixed distance and flew in sequence during the charging period. Figure 9 shows the comparison of the average charging time for the drones on ordinary missions and the drones on time-critical missions. It can be observed that the extra time spent on charging by a drone on the ordinary mission was significantly higher during peak periods owing to the congestion that occurred at the traditional fix-point wireless charging and battery swap stations. However, the curve for the time spent on charging by a drone on a time-critical mission is flatter than that for the number of charging requests as shown in Figure 6. Although the path of the drone on the time-critical mission does not exactly match the preplanned flight path if the distributed laser charging option was chosen, the time spent on charging for a drone was lower, even if it might deviate slightly from the original flight path. In summary, the time spent on charging a drone on a time-critical mission is much lower when compared with that for a drome on an ordinary mission. That is, it is much better for the drones on time-critical missions to adopt distributed laser charging facility or drone-to-drone charging options than queuing up at the traditional fixed-point wireless charging stations and battery swap stations during peak hours.

**Figure 10:**
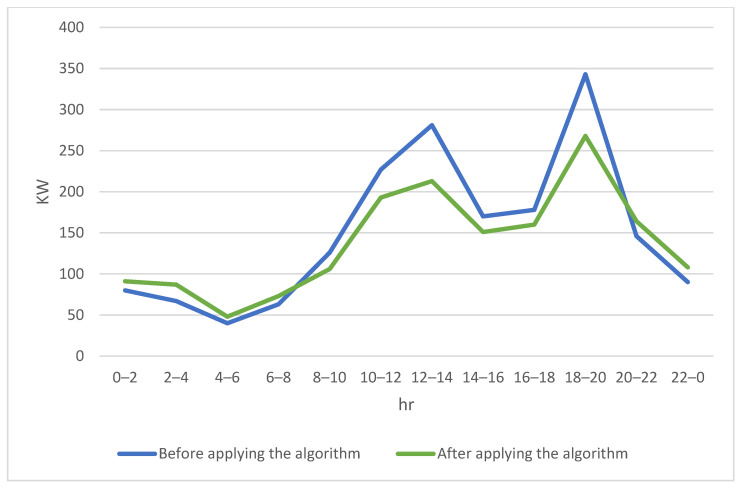
Power demand at fixed-point wireless charging stations and battery swap stations.

Figure 10 shows the comparison of power demand of fixed-point wireless charging stations and battery swap stations. This work assumes a drone got charged after it arrived at the apron to refill the power with cheaper electricity. It can be seen that the power supply to the requesting drones during morning and evening peak hours is higher before the application of the proposed work. Accordingly, the congestion for drones waiting for charging that occurred at the traditional fixed-point wireless charging stations and battery swap stations was alleviated, and the power load was also mitigated via distributed laser charging facilities or drone-to-drone charging during peak periods. 

## 4. Conclusions

Although few studies have proposed algorithms to provide power to drones with urgent needs through wireless charging technology, the presented charging schemes in the literature are oversimplified and have many restrictions. In addition, different charging options should be offered for each individual drone depending on its mission characteristics and needs. In view of this, this work proposed a joint routing path and charging plan for EVs and drones to meet the task characteristics and charging needs of various types of drones. The experimental results demonstrated that the flight path and charging mechanism proposed in this study can effectively reduce the time spent on charging when the drones perform time-critical missions at peak times of delivery orders, and enable the drone to deliver urgent goods to the designated destination on schedule even when the traditional fixed-point wireless charging stations and battery swap stations are congested. Therefore, the proposed algorithm can not only reduce the congestion at fixed-point charging stations or battery swap services during peak hours, it will also help drones with charging needs to arrive at their destinations in time to complete their missions during peak hours and improve the imbalance between power supply and demand in the power grid due to the weather conditions and lower than expected green power supply. New and advanced charging technologies for drones have been proposed by researchers. In future work, we will incorporate evolving charging technologies proposed by researchers into our integrated flight path and charging mechanism to meet the task characteristics and charging needs of various types of drones.

## Data Availability

Not applicable.
